# Pseudo Numerical Ranges and Spectral Enclosures

**DOI:** 10.1007/s11785-022-01232-9

**Published:** 2022-06-30

**Authors:** Borbala Gerhat, Christiane Tretter

**Affiliations:** Math. Institut, Universität Bern, Sidlerstr. 5, 3012 Bern, Switzerland

**Keywords:** Numerical range, Spectrum, Resolvent estimate, Operator function, Operator polynomial, Damped wave equation

## Abstract

We introduce the new concepts of pseudo numerical range for operator functions and families of sesquilinear forms as well as the pseudo block numerical range for $n\!\times \! n$ operator matrix functions. While these notions are new even in the bounded case, we cover operator polynomials with unbounded coefficients, unbounded holomorphic form families of type (a) and associated operator families of type (B). Our main results include spectral inclusion properties of pseudo numerical ranges and pseudo block numerical ranges. For diagonally dominant and off-diagonally dominant operator matrices they allow us to prove spectral enclosures in terms of the pseudo numerical ranges of Schur complements that no longer require dominance order 0 and not even $<\!1$. As an application, we establish a new type of spectral bounds for linearly damped wave equations with possibly unbounded and/or singular damping.

## Introduction

Spectral problems depending non-linearly on the eigenvalue parameter arise frequently in applications, see e.g. the comprehensive collection in [2] or the monograph [21]. The dependence ranges from quadratic in problems originating in second order Cauchy problems such as damped wave equations, see e.g. [13, 15], to rational as in electromagnetic problems with frequency dependent materials such as photonic crystals, see e.g. [9, 1]. In addition, if energy dissipation is present due to damping or lossy materials, then the values of the corresponding operator functions need not be selfadjoint.

While for operator functions $T(\uplambda )$, $\uplambda \!\in \!\Omega \!\subseteq \! {\mathbb {C}}$, with unbounded operator values in a Hilbert space ${\mathcal {H}}$ the notion of numerical range *W*(*T*) exists,

1.1 $$\begin{aligned} \begin{aligned} W(T)\!:=&\!\left\{ \uplambda \!\in \!\Omega :0\!\in \! W(T(\uplambda ))\right\} \\ \!=&\left\{ \uplambda \!\in \!\Omega :\exists \, f\!\in \!{\text {dom}}T(\uplambda ), f\!\ne \!0, \ (T(\uplambda )f,f)\!=\!0\right\} , \end{aligned} \end{aligned}$$

a spectral inclusion result $\sigma _{\mathrm{ap}}(T) \!\subseteq \! \overline{W(T)} \cap \Omega$ for the approximate point spectrum is lacking. Even in the case of bounded values $T(\uplambda )$, spectral inclusion only holds under a certain condition that is not easy to verify. Moreover, spectral inclusion results are even lacking for the most important case of quadratic operator polynomials with unbounded coefficients, one of the most relevant cases for applications.

In the present paper we fill these gaps. To this end, we introduce the novel concept of *pseudo numerical range* of operator functions $T(\uplambda )$, $\uplambda \!\in \!\Omega \!\subseteq \! {\mathbb {C}}$, with unbounded values,

$$\begin{aligned} W_\Psi (T){:}{=}\bigcap \limits _{\varepsilon>0}W_\varepsilon (T), \quad W_\varepsilon (T){:}{=}\bigcup \limits _{\begin{array}{c} B\in L({\mathcal {H}}) \\ \left\Vert B\right\Vert <\varepsilon \end{array}}W(T+B), \quad \varepsilon >0, \end{aligned}$$

and analogously for families of unbounded quadratic forms ${\mathbf {t}}(\uplambda )$, $\uplambda \!\in \!\Omega \!\subseteq \! {\mathbb {C}}$. The sets $W_\varepsilon (T)$, $\varepsilon >0$, can be shown to have the equivalent form

$$\begin{aligned} W_\varepsilon (T) =\left\{ \uplambda \in \Omega :\exists ~f\in {\text {dom}}T(\uplambda ), ~\left\Vert f\right\Vert =1, ~\left|(T(\uplambda )f,f)\right|<\varepsilon \right\} ; \end{aligned}$$

hence they coincide with the so-called $\varepsilon$-pseudo numerical range first considered in [10]. As a consequence, the pseudo numerical range $W_\Psi (T)$ can equivalently be described as

1.2 $$\begin{aligned} W_\Psi (T) \!=\!\big \{\uplambda \!\in \!\Omega :0\!\in \!\overline{W(T(\uplambda ))}\big \} {=}{:}W_{\Psi ,0}(T). \end{aligned}$$

One could be tempted to think that the condition $0\!\in \!\overline{W(T(\uplambda ))}$ in $W_{\Psi ,0}(T)$ is equivalent to $\uplambda \!\notin \! \overline{W(T)}$, but this is neither true for operator functions with bounded values, as already noted in [31], nor for non-monic linear operator pencils for which the set $W_{\Psi ,0}(T)$ was used recently in [3].

One of the crucial properties of the pseudo numerical range is that, *without any assumptions on the operator family*,

$$\begin{aligned} \sigma _{{\text {ap}}}(T)\subseteq W_\Psi (T), \end{aligned}$$

see Theorem 3.1, and that the norm of the resolvent of *T* can be estimated by

$$\begin{aligned} \left\Vert T(\uplambda )^{-1}\right\Vert \le \varepsilon ^{-1}, \quad \uplambda \in \rho (T){\setminus } W_\varepsilon (T) \subseteq \rho (T){\setminus } W_\Psi (T). \end{aligned}$$

Not only from the analytical point of view, but also from a computational perspective, the pseudo numerical range seems to be more convenient since it is much easier to determine whether a number is small rather than zero.

Like the numerical range of an operator function, but in contrast to the numerical range or essential numerical range of an operator [4, 12, 17], the pseudo numerical range need not be convex. An exception is the trivial case of a monic linear operator pencil $T(\uplambda )\!=\!A\!-\!\uplambda I$, $\uplambda \!\in \! {\mathbb {C}}$, where the pseudo numerical range is simply the closure of the numerical range, $W_\Psi (T)\!=\!\overline{W(T)}\!=\!\overline{W(A)}$. In general, we only have the obvious enclosure $W(T) \subseteq W_\Psi (T)$. Neither the interiors nor the closures in $\Omega$ of $W_\Psi (T)$ and *W*(*T*) need to coincide and there is also no inclusion either way between $W_\Psi (T)$ or its closure $\overline{W_\Psi (T)}\cap \Omega$ in $\Omega$ and the closure $\overline{W(T)} \cap \Omega$ of *W*(*T*) in $\Omega$; we give various counter-examples to illustrate these effects.

In our first main result we use the pseudo numerical range of holomorphic form families ${\mathbf {t}}(\uplambda )$, $\uplambda \in \Omega$, of type (a) to prove the spectral inclusion for the associated holomorphic operator functions $T(\uplambda )$, $\uplambda \in \Omega$, of type (B) of m-sectorial operators $T(\uplambda )$. More precisely, we show that if there exist $k\in {\mathbb {N}}_0$, $\mu \in \Omega$ and a core ${\mathcal {D}}$ of ${\mathbf {t}}(\mu )$ with

1.3 $$\begin{aligned} 0 \notin \overline{W\big ({\mathbf {t}}^{(k)}(\mu )\big |_{\mathcal {D}}\big )}, \end{aligned}$$

then $\sigma (T) \subseteq W_\Psi ({\mathbf {t}})=\overline{W({\mathbf {t}})} \cap \Omega$ and, if in addition, the operator family *T* has constant domain, then

1.4 $$\begin{aligned} \sigma (T) \!\subseteq \, W_\Psi (T)=\overline{W(T)}\cap \Omega , \end{aligned}$$

see Theorem 3.3. Note that, due to (1.2), condition (1.3) for $k\!=\!0$, i.e. $0 \notin \overline{W\big ({\mathbf {t}}(\mu )\big |_{\mathcal {D}}\big )}$ for some $\mu \in {\mathbb {C}}$, is equivalent to $W_\Psi (T)\ne \Omega$.

For operator polynomials $T(\uplambda ) = \sum _{k=0}^n \uplambda ^k A_k$ with domain ${\text {dom}}T(\uplambda )=\bigcap _{k=0}^n {\text {dom}}A_k$, $\uplambda \in {\mathbb {C}}$, we prove that, if $0\notin \overline{W(A_n)}$, then

$$\begin{aligned} \sigma _{{\text {ap}}}(T) \subseteq W_\Psi (T)\subseteq \overline{W(T)}\cap \Omega , \end{aligned}$$

see Proposition 2.7. The inclusion (1.4) follows if, in addition, $\sigma (T(\uplambda )) \!\subseteq \! \overline{W(T(\uplambda ))}$, $\uplambda \!\in \!{\mathbb {C}}$, which is a weaker condition than m-sectoriality of all $T(\uplambda )$.

The second new concept we introduce in this paper is the *pseudo block numerical range* of operator functions ${\mathcal {L}}(\uplambda )$, $\uplambda \in \Omega$, that possess an operator matrix representation with respect to a decomposition ${\mathcal {H}}={\mathcal {H}}_1\oplus \cdots \oplus {\mathcal {H}}_n$, $n\in {\mathbb {N}}$, of the given Hilbert space ${\mathcal {H}}$. This means that

$$\begin{aligned} {\mathcal {L}}(\uplambda )=\big ( L_{ij} (\uplambda ) \big )_{i,j=1}^n, \quad {\text {dom}}{\mathcal {L}}(\uplambda )=\bigoplus \limits _{\!j=1}^{\!n} \ \bigcap \limits _{i=1}^n {\text {dom}}L_{ij}(\uplambda ), \end{aligned}$$

with operator functions $L_{ij}(\uplambda )$, $\uplambda \!\in \!\Omega$, of densely defined and closable linear operators from ${\mathcal {H}}_j$ to ${\mathcal {H}}_i$, *i*, $j=1,\dots , n$.

Extending earlier concepts we first define the *block numerical range* of ${\mathcal {L}}$ as

$$\begin{aligned} W^{n}({\mathcal {L}}) {:}{=}\bigcup \limits _{\begin{array}{c} (f_i)\in {\text {dom}}{\mathcal {L}}(\uplambda ) \\ \Vert f_i\Vert \!=\!1 \end{array}} \sigma _p \big ( {\mathcal {L}}(\uplambda )_{(f_i)} \big ), \quad {\mathcal {L}}(\uplambda )_{(f_i)} \!{:}{=}\! \left( {\mathcal {L}}_{ij}(\uplambda ) f_j, f_i \right) \!\in \! {\mathbb {C}}^{n\times n}\!; \end{aligned}$$

for bounded values ${\mathcal {L}}(\uplambda )$ see [23] and [28] for $n=2$, for unbounded operator matrices ${\mathcal {L}}(\uplambda ) \!=\! {{\mathcal {A}}} - \uplambda I_{\mathcal {H}}$ see [24]. Then we introduce the *pseudo block numerical range* of ${\mathcal {L}}$ as

$$\begin{aligned} W^n_\Psi ({\mathcal {L}}){:}{=}\bigcap \limits _{\varepsilon>0}W_\varepsilon ^n({\mathcal {L}}), \qquad W_\varepsilon ^n({\mathcal {L}}){:}{=}\bigcup \limits _{\begin{array}{c} {\mathcal {B}}\in L({\mathcal {H}})\\ \left\Vert {\mathcal {B}}\right\Vert <\varepsilon \end{array}} W^n({\mathcal {L}}+{\mathcal {B}}), \quad \varepsilon >0. \end{aligned}$$

For $n\!=\!1$ both block numerical range and pseudo block numerical range coincide with the numerical range and pseudo numerical range of ${\mathcal {L}}$, respectively. For $n\!>\!1$, the trivial inclusion $W^n({\mathcal {L}}) \subseteq W^n_\Psi ({\mathcal {L}})$ and the characterisation (1.1), i.e.

$$\begin{aligned} W^n({\mathcal {L}}) =\big \{ \uplambda \in \Omega : 0\in W^n({\mathcal {L}}(\uplambda )) \big \}, \quad n\in {\mathbb {N}}, \end{aligned}$$

and a resolvent norm estimate

$$\begin{aligned} \left\Vert {\mathcal {L}}(\uplambda )^{-1}\right\Vert \!\le \! \varepsilon ^{-1}, \quad \uplambda \!\in \!\rho ({\mathcal {L}}){\setminus } W_\varepsilon ^{n}({\mathcal {L}}) \subseteq \! \rho ({\mathcal {L}}){\setminus } W_\Psi ^n({\mathcal {L}}), \quad n\!\in \!{\mathbb {N}}, \end{aligned}$$

see Theorem 4.10 for both, continue to hold, but otherwise not much carries over from the case $n\!=\!1$. The first difference is that, for the simplest case ${\mathcal {L}}(\uplambda )={\mathcal {A}}-\uplambda I_{\mathcal {H}}$, $\uplambda \in {\mathbb {C}}$, we may have $W_\Psi ^n({\mathcal {L}})\ne \overline{W^n({\mathcal {L}})}$ for $n\!>1\!$, see Example 4.5.

More importantly, for $n\!>\!1$ the relation (1.2) need not hold for the pseudo block numerical range; here we only have the inclusion

$$\begin{aligned} W_\Psi ^{n}({\mathcal {L}}) \supseteq \left\{ \uplambda \!\in \!\Omega :0\in \overline{W^{n}({\mathcal {L}}(\uplambda ))}\right\} {=}{:}W_{\Psi ,0}^{n}({\mathcal {L}}), \quad n\in {\mathbb {N}}, \end{aligned}$$

see Proposition 4.4. Therein we also assess two other candidates $W_{\Psi ,i}^{n}({\mathcal {L}}) \!=\! \bigcap _{\varepsilon >0} W_{\varepsilon ,i}^{n}({\mathcal {L}})$, $i\!=\!1,2$, for the pseudo block numerical range for which $W_{\varepsilon ,1}^{n}({\mathcal {L}})$ is defined by the scalar condition $\det {\mathcal {L}}(\uplambda )_{(f_i)} \!<\! \varepsilon$ and $W_{\varepsilon ,2}^{n}({\mathcal {L}})$ by restricting to *diagonal* perturbations ${\mathcal {B}}\in L({\mathcal {H}})$ with $\left\Vert {\mathcal {B}}\right\Vert <\varepsilon$. In fact, we show that

1.5 $$\begin{aligned} W^n({\mathcal {L}}) \subseteq W^{n}_{\Psi ,1}({\mathcal {L}})\subseteq W_{\Psi ,0}^{n}({\mathcal {L}})\subseteq W^{n}_{\Psi ,2}({\mathcal {L}})\subseteq W^{n}_\Psi ({\mathcal {L}}), \end{aligned}$$

and that, like the pseudo numerical range, the pseudo block numerical range $W^{n}_\Psi ({\mathcal {L}})$ has the spectral inclusion property, i.e.

$$\begin{aligned} \sigma _{{\text {ap}}}(T) \subseteq W^{n}_\Psi ({\mathcal {L}}) \subseteq W_\Psi (T), \quad n\in {\mathbb {N}}, \end{aligned}$$

but, in general, none of the subsets of $W^{n}_\Psi ({\mathcal {L}})$ in (1.5) is large enough to contain $\sigma _{{\text {ap}}}(T)$, see Example 4.5.

Our second main result concerns the most important case $n\!=\!2$, the so-called *quadratic numerical range* and *pseudo quadratic numerical range*. Here we prove a novel type of spectral inclusion for diagonally dominant and off-diagonally dominant ${\mathcal {L}}(\uplambda ) \!=\! (L_{ij}(\uplambda ))_{i,j=1}^2$ in terms of the pseudo numerical ranges of the Schur complements $S_1$, $S_2$ and, further, the pseudo quadratic numerical range of ${\mathcal {L}}$,

$$\begin{aligned} \sigma _{{\text {ap}}}({\mathcal {L}}){\setminus }(\sigma (L_{11})\cup \sigma (L_{22})) \subseteq W_\Psi (S_1)\cup W_\Psi (S_2) \subseteq W^2_\Psi ({\mathcal {L}}), \end{aligned}$$

see Theorem 5.1, where $S_1(\uplambda )\!=\!L_{11}(\uplambda ) \!-\! L_{12}(\uplambda ) L_{22}(\uplambda )^{-1} L_{21}(\uplambda )$, $\uplambda \!\in \! \rho (L_{22})$, and similarly for $S_2$ with the indices 1 and 2 reversed. For symmetric and anti-symmetric corners, i.e. $L_{21}(\uplambda ) \subseteq \pm L_{12}(\uplambda )^*$, $\uplambda \!\in \!\Omega$, we even show that

$$\begin{aligned} \sigma _{{\text {ap}}}({\mathcal {L}}) \!\subseteq \! W_\Psi (S_1)\cup W_\Psi (L_{22}), \end{aligned}$$

if $L_{11}(\uplambda )$ is accretive, ${\mp } L_{22}(\uplambda )$ is m-sectorial and ${\text {dom}}L_{22}(\uplambda ) \!\subseteq \! {\text {dom}}L_{12}(\uplambda )$, see Theorem 5.3/Corollary 5.4, and similarly for the Schur complement $S_2$.

As an interesting consequence, we are able to establish spectral separation and inclusion theorems for unbounded $2\!\times \!2$ operator matrices ${\mathcal {A}}=(A_{ij})_{i,j=1}^2$ with ’separated’ diagonal entries; here ’separated’ means that the numerical ranges of $A_{11}$ and $A_{22}$ lie in half-planes and/or sectors in the right and left half-plane ${\mathbb {C}}_+$ and ${\mathbb {C}}_-$, respectively, separated by a vertical strip $S\!{:}{=}\!\{z\!\in \!{\mathbb {C}}:\delta \!< \! {\text {Re}}z \!< \! \alpha \}$ with $\delta \!<\!0\!<\!\alpha$ around $\mathrm{{i}}{\mathbb {R}}$. More precisely, *without* any bounds on the order of diagonal dominance or off-diagonal dominance we show that, if $\varphi$, $\psi \!\in \! [0,\frac{\pi }{2}]$ are the semi-angles of $A_{11}$ and $A_{22}$ and $\tau \!:=\! \max \{\varphi ,\psi \}$, then

$$\begin{aligned} \sigma _{{\text {ap}}}({\mathcal {A}}) \subseteq ( - \!\Sigma _\tau \cup \Sigma _\tau ) {\setminus } S {=}{:}\Sigma , \quad \Sigma _\tau {:}{=}\{z\!\in \!{\mathbb {C}}: |\arg z| \le \tau \}, \end{aligned}$$

and $\sigma ({\mathcal {A}}) \subseteq \Sigma$ if $\rho ({\mathcal {A}}) \cap ({\mathbb {C}}{\setminus } \Sigma ) \!\ne \! \emptyset$, see Theorem 6.1. This result is a great step ahead compared to the earlier result [27,  Thm. 5.2] where the dominance order had to be restricted to 0.

Moreover, even to ensure the condition $\rho ({\mathcal {A}}) \cap ({\mathbb {C}}{\setminus } \Sigma ) \!\ne \! \emptyset$ for the enclosure of the entire spectrum $\sigma ({\mathcal {A}})$ in Theorem 6.1, we do not have to restrict the dominance order as usual for perturbation arguments. Our new weak conditions involve only products of the columnwise relative bounds $\delta _1$ in the first and $\delta _2$ in the second column, see Proposition 6.5; in particular, either $\delta _1\!=\!0$ or $\delta _2\!=\!0$ guarantees $\rho ({\mathcal {A}}) \cap ({\mathbb {C}}{\setminus } \Sigma ) \!\ne \! \emptyset$ in Theorem 6.1 and hence $\sigma _{{\text {ap}}}({\mathcal {A}}) \!\subseteq \! \Sigma$.

As an application of our results, we consider abstract quadratic operator polynomials $T(\uplambda )$, $\uplambda \!\in \!{\mathbb {C}}$, induced by forms ${\mathbf {t}}(\uplambda )\!=\!{\mathbf {t}}_0\!+\!2\uplambda {\mathbf {a}}\!+\!\uplambda ^2$ with ${\text {dom}}{\mathbf {t}}(\uplambda )={\text {dom}}{\mathbf {t}}_0$, $\uplambda \in {\mathbb {C}}$, as they arise e.g. from linearly damped wave equations

1.6 $$\begin{aligned} u_{tt}(x,t)+2a(x)u_t(x,t)=\left( \Delta _x-q(x)\right) u(x,t), \quad x\in {\mathbb {R}}^d, \quad t>0, \end{aligned}$$

where the non-negative potential *q* and damping *a* may be singular and/or unbounded, cf. [11, 13–15] where also accretive damping was considered, and for which it is well-known that the spectrum is symmetric with respect to ${\mathbb {R}}$ and confined to the closed left half-plane.

Here we use a finely tuned assumption on the ’unboundedness’ of ${\mathbf {a}}$ with respect to ${\mathbf {t}}_0$, namely *p*-*subordinacy* for $p\!\in \![0,1)$, comp. [20,  § 5.1] or [29,  Sect. 3] for the operator case. More precisely, if ${\mathbf {t}}_0\!\ge \!\kappa _0\!\ge \!0$, ${\mathbf {a}}\!\ge \!\alpha _0\!\ge \!0$ with ${\text {dom}}{\mathbf {t}}_0\!\subseteq \!{\text {dom}}{\mathbf {a}}$ and there exist $p\!\in \![0,1)$ and $C_{p}\!>\!0$ with

$$\begin{aligned} {\mathbf {a}}[f]\le C_{p}\big ({\mathbf {t}}_0[f]\big )^p \big (\left\Vert f\right\Vert ^2\big )^{1-p}, \quad f\in {\text {dom}}{\mathbf {t}}_0, \end{aligned}$$

we use the enclosure $\sigma (T) \!\subseteq \! W_\Psi (T) \!=\! W_\Psi ({\mathbf {t}}) \!=\! \overline{W({\mathbf {t}})}$ to prove that the non-real spectrum of *T* satisfies the bounds

$$\begin{aligned} \sigma (T){\setminus } {\mathbb {R}}\! \subseteq \,&\! \Big \{ z\!\in \!{\mathbb {C}}: \, |z| \ge \sqrt{\kappa _0}, \, \, {\text {Re}}z\le -\alpha _0,\\&\, \left|{\text {Im}}z\right|^2\!\!\ge \! \max \!\big \{0,C_{p}^{-\frac{1}{p}}\!\left|{\text {Re}}z\right|^\frac{1}{p}\!\!-\!\left|{\text {Re}}z\right|^2\big \}\Big \} \end{aligned}$$

and the real spectrum $\sigma (T)\cap {\mathbb {R}}\subset [-\infty ,0]$ is either empty or it is confined to one bounded interval, to one unbounded interval or to the disjoint union of a bounded and an unbounded interval , see Theorem 7.1 and Figure 2. Moreover, we describe both the thresholds for the transitions between these cases and the enclosures for $\sigma (T)\cap {\mathbb {R}}$ precisely in terms of *p*, $C_p$, $\kappa$ and $\kappa _0$. As a concrete example, we consider the damped wave equation (1.6) with

$$\begin{aligned} a(x)\!\le \!\sum _{j=1}^n\left|x\!-\!x_j\right|^{-t}\!+\! u(x) \!+\! v(x), \ \ v(x) \!\le \! c_1 q(x)^r\!+c_2 \,\ \text{ for } \text{ almost } \text{ all } x\!\in \! {\mathbb {R}}^d, \end{aligned}$$

where $n\!\in \!{\mathbb {N}}_{0}$, $x_j\!\in \!{\mathbb {R}}^d$ for $j\!=\!1,\dotsc ,n$, $u \!\in \! L^s ({\mathbb {R}}^d)$ with $s\!>\!\frac{d}{2}$, $v\!\in \!L^1_{{\text {loc}}}({\mathbb {R}}^d)$, $t\!\in \![0,2)$, $c_1$, $c_2\!\ge \!0$ and $r\!\in \![0,1)$. For the special case $q(x)\!=\!\left|x\right|^2$, $a(x)\!=\!\left|x\right|^{k}$, $x\!\in \!{\mathbb {R}}^d$, with $k \!\in \![0,2)$, the new spectral enclosure in Theorem 7.1 yields

$$\begin{aligned} \sigma (T) {\setminus }{\mathbb {R}}\subseteq \Big \{z\!\in \!{\mathbb {C}}:{\text {Re}}z\!\le \!0, \, \left|z\right|\!\ge \! \sqrt{d}, \, |{\text {Im}}z| \!\ge \! \sqrt{\max \{0,\left|{\text {Re}}z\right|^{\!\frac{2}{k} }\!\!-\!\left|{\text {Re}}z\right|^2\}}\Big \} \end{aligned}$$

and, with $$t_0=\max \big \{ \big ( k(2-k) \big )^{-\frac{1}{k-1}},d\big \}$$,

$$\begin{aligned} \sigma (T) \cap {\mathbb {R}}{\left\{ \begin{array}{ll} = \emptyset &{} \text{ if } k\!\in \![0,1), \\ \subseteq (-\infty ,-\sqrt{d}] &{} \text{ if } k=1, \\ \subseteq \!\Big (\!\!-\!\infty ,-\sqrt{t_0}^{k}\!+\!\sqrt{t_0^{k}\!-\!t_0 } \,\Big ] &{} \text{ if } k\!\in \! (1,2). \end{array}\right. } \end{aligned}$$

The paper is organised as follows. In Sect. 2 we introduce the pseudo numerical range of operator functions and form functions and study the relation of $W_\Psi (T)$ and $\overline{W(T)}\cap \Omega$. In Sect. 3 we establish spectral inclusion results in terms of the pseudo numerical range. In Sect. 4 we define the block numerical range $W^n({\mathcal {L}})$ and pseudo block numerical range $W^{n}_\Psi ({\mathcal {L}})$ of unbounded $n\!\times \! n$ operator matrix functions ${\mathcal {L}}$, investigate the differences to the special case $n\!=\!1$ of the pseudo numerical range $W_\Psi ^1({\mathcal {L}})\!=\!W_\Psi ({\mathcal {L}})$ and prove corresponding spectral inclusion theorems. In Sect. 5 we establish new enclosures of the approximate point spectrum of $2\!\times \! 2$ operator matrix functions by means of the pseudo numerical ranges of their Schur complements. In Sect. 6 we apply them to prove spectral bounds for diagonally dominant and off-diagonally dominant operator matrices with symmetric or anti-symmetric corners without restriction on the dominance order. Finally, in Sect. 7, we apply our results to linearly damped wave equations with possibly unbounded and/or singular damping and potential.

Throughout this paper, ${\mathcal {H}}$ and ${\mathcal {H}}_i$, $i\!=\!1,\dots ,n$, denote Hilbert spaces, $L({\mathcal {H}})$ denotes the space of bounded linear operators on ${\mathcal {H}}$ and $\Omega \!\subseteq \!{\mathbb {C}}$ is a domain.

## The Pseudo Numerical Range of Operator Functions and Form Functions

In this section, we introduce the new notion of pseudo numerical range for operator functions $\left\{ T(\uplambda ):\uplambda \in \Omega \right\}$ and form functions $\left\{ {\mathbf {t}}(\uplambda ):\uplambda \in \Omega \right\}$, respectively, briefly denoted by *T* and ${\mathbf {t}}$ if no confusion about $\Omega$ can arise. While the values $T(\uplambda )$ and ${\mathbf {t}}(\uplambda )$ may be bounded/unbounded linear operators and sesquilinear forms in a Hilbert space ${\mathcal {H}}$, the notion of pseudo numerical range is new also in the bounded case.

The *numerical range* of *T* and ${\mathbf {t}}$, respectively, are defined as

$$\begin{aligned} W(T)\!&=\!\left\{ \uplambda \!\in \!\Omega \!:\!0\!\in \! W(T(\uplambda ))\right\} =\!\left\{ \uplambda \!\in \!\Omega \!:\!\exists \, f\!\in \!{\text {dom}}T(\uplambda ), f\!\ne \!0, (T(\uplambda )f,f)\!=\!0\right\} , \\ W({\mathbf {t}})\!&=\!\left\{ \uplambda \!\in \!\Omega \!:\!0\!\in \! W({\mathbf {t}}(\uplambda ))\right\} \!=\!\left\{ \uplambda \!\in \!\Omega \!:\!\exists \, f\!\in \!{\text {dom}}{\mathbf {t}}(\uplambda ), \,f\!\ne \!0, \,{\mathbf {t}}(\uplambda )[f]\!=\!0\right\} , \end{aligned}$$

comp. [20,  § 26]. In the simplest case of a monic linear operator polynomial $T(\uplambda ) = T_0 - \uplambda I_{\mathcal {H}}$, $\uplambda \in {\mathbb {C}}$, this notion coincides with the numerical range $W(T_0)$ of the linear operator $T_0$, and analogously for forms; note that the latter is also denoted by $\Theta (T_0)$, e.g. in [17,  Sect. V.3.2].

The following new concept of pseudo numerical range employs the notion of $\varepsilon$-pseudo numerical range $W_\varepsilon (T)$, $\varepsilon >0$, introduced in [10,  Def. 4.1]; the equivalent original definition therein, see (2.1) below, was designed to obtain computable enclosures for spectra of rational operator functions.

### Definition 2.1

We introduce the *pseudo numerical range* of an operator function *T* and a form function ${\mathbf {t}}$, respectively, as

$$\begin{aligned} \begin{aligned} W_\Psi (T)&{:}{=}\bigcap _{\varepsilon>0}W_\varepsilon (T),&\quad W_\Psi ({\mathbf {t}})&{:}{=}\bigcap _{\varepsilon >0}W_\varepsilon ({\mathbf {t}}),\\ \end{aligned} \end{aligned}$$

where

$$\begin{aligned} W_\varepsilon (T) {:}{=}\bigcup _{B \in L({\mathcal {H}}), \left\Vert B\right\Vert<\varepsilon }W(T+B), \quad W_\varepsilon ({\mathbf {t}}) {:}{=}\bigcup _{\left\Vert {\mathbf {b}}\right\Vert <\varepsilon }W({\mathbf {t}}+{\mathbf {b}}), \quad \varepsilon >0; \end{aligned}$$

here $\left\Vert {\mathbf {b}}\right\Vert =\sup _{\left\Vert f\right\Vert =\left\Vert g\right\Vert =1}\left|{\mathbf {b}}[f,g]\right|$ for a bounded sesquilinear form ${\mathbf {b}}$ in ${\mathcal {H}}$.

Clearly, for monic linear operator polynomials $T(\uplambda ) = A - \uplambda I_{\mathcal {H}}$, $\uplambda \in {\mathbb {C}}$, the pseudo numerical range is nothing but the closure of the classical numerical range $\overline{W(A)}$ of the linear operator *A*, and analogously for forms.

The pseudo numerical range of operator or form functions, is, like their numerical ranges, in general neither convex nor connected, and, even for families of bounded operators or forms, it may be unbounded.

### Remark 2.2

(i) The following enclosures may be proper, see Example 3.2,
  $$\begin{aligned} W(T)\subseteq W_\Psi (T), \qquad W({\mathbf {t}})\subseteq W_\Psi ({\mathbf {t}}). \end{aligned}$$
(ii) In general, the pseudo numerical range need neither be open nor closed in $\Omega$ equipped with the relative topology, see Examples 3.2 (i) and 2.9, respectively.
(iii) Neither the closures nor the interiors with respect to the relative topology on $\Omega$ of the pseudo numerical range and the numerical range need to coincide, see Example 3.2 (i) and (ii).

The following alternative characterisation of the pseudo numerical range will be frequently used in the sequel.

### Proposition 2.3

For every $$\varepsilon >0$$,

2.1 $$\begin{aligned} W_\varepsilon (T)&=\left\{ \uplambda \in \Omega :\exists ~f\in {\text {dom}}T(\uplambda ), ~\left\Vert f\right\Vert =1, ~\left|(T(\uplambda )f,f)\right|<\varepsilon \right\} , \nonumber \\ W_\varepsilon ({\mathbf {t}})&=\left\{ \uplambda \in \Omega :\exists ~f\in {\text {dom}}{\mathbf {t}}(\uplambda ), ~\left\Vert f\right\Vert =1, ~\left|{\mathbf {t}}(\uplambda )[f]\right|<\varepsilon \right\} , \end{aligned}$$

and, consequently,

2.2 $$\begin{aligned} W_\Psi (T)&\!=\!\left\{ \uplambda \!\in \!\Omega :0\!\in \!\overline{W(T(\uplambda ))}\right\} , \ \ W_\Psi ({\mathbf {t}}) \!=\!\left\{ \uplambda \!\in \!\Omega :0\!\in \!\overline{W({\mathbf {t}}(\uplambda ))}\right\} . \end{aligned}$$

### Proof

We show the claim for $W_\varepsilon (T)$; then the claim for $W_\Psi (T)$ is obvious by Definition 2.1. The proof for $W_\varepsilon ({\mathbf {t}})$ and $W_\Psi ({\mathbf {t}})$ is analogous.

Let $\varepsilon >0$ be arbitrary and $\uplambda \in W_\varepsilon (T)$. There exists a bounded operator *B* in ${\mathcal {H}}$ with $\left\Vert B\right\Vert <\varepsilon$ such that $\uplambda \in W(T+B)$, i.e.

$$\begin{aligned} \left( T(\uplambda )f,f\right) =-(Bf,f), \quad f\in {\text {dom}}T(\uplambda ), \quad \left\Vert f\right\Vert =1. \end{aligned}$$

Hence, clearly, $\left|\left( T(\uplambda )f,f\right) \right|\le \left\Vert B\right\Vert <\varepsilon$, thus $\uplambda$ is an element of the right hand side of (2.1).

Conversely, let $\uplambda \in \Omega$ such that there exists $f\in {\text {dom}}T(\uplambda )$, $\left\Vert f\right\Vert =1$, with $\left|\left( T(\uplambda )f,f\right) \right|<\varepsilon$. Setting $B{:}{=}-\left( T(\uplambda )f,f\right) I$, this gives $\uplambda \in W(T+B)$ and $\left\Vert B\right\Vert =\left|\left( T(\uplambda )f,f\right) \right|<\varepsilon$, hence $\uplambda \in W_\varepsilon (T)$. $\square$

The following properties of the pseudo numerical range with respect to closures, form representations and Friedrichs extensions are immediate consequences of its alternative description (2.2).

Here an operator *A* or a form ${\mathbf {a}}$ is called *sectorial* if its numerical range lies in a sector $\{z{\in }{\mathbb {C}}: |\arg (z-\gamma )| {\le } \vartheta \}$ for some $\gamma {\in }{\mathbb {R}}$ and $\vartheta {\in } [0, \frac{\pi }{2})$, see [17,  Sect. V.3.10, VI.1.2]; if, in addition, $\rho (A) \cap \{z \in {\mathbb {C}}:|\arg (z-\gamma )| > \vartheta \} \ne \emptyset$, then *A* is called m-sectorial.

### Corollary 2.4

(i) If the family *T* or ${\mathbf {t}}$, respectively, consists of closable operators or forms (and ${\overline{T}}$ or ${\overline{{\mathbf {t}}}}$ denotes the family of closures), then
  $$\begin{aligned} W_\Psi (T)=W_\Psi ({\overline{T}}), \qquad W_\Psi ({\mathbf {t}})=W_\Psi ({\overline{{\mathbf {t}}}}). \end{aligned}$$
(ii) If the family ${\mathbf {t}}$ consists of densely defined closed sectorial forms and *T* denotes the family of associated m-sectorial operators, then
  $$\begin{aligned} W_\Psi ({\mathbf {t}})=W_\Psi (T). \end{aligned}$$
(iii) If the family *T* consists of densely defined sectorial operators and $T_F$ denotes the family of corresponding Friedrichs extensions then
  $$\begin{aligned} W_\Psi (T)=W_\Psi (T_F). \end{aligned}$$

### Proof

(i) The equalities follow from Proposition 2.3 and from the fact that $\overline{W(T(\uplambda ))}=\overline{W(\overline{T(\uplambda )})}$ and $\overline{W({\mathbf {t}}(\uplambda ))}=\overline{W({\overline{{\mathbf {t}}}}(\uplambda ))}$ for $\uplambda \in \Omega$, see [17,  Prob. V.3.7, Thm. VI.1.18].

(ii) The equality follows from Proposition 2.3 and the identity $\overline{W({\mathbf {t}}(\uplambda ))}=\overline{W(T(\uplambda ))}$ for $\uplambda \in \Omega$, see [17,  Cor. VI.2.3].

(iii) The claim is a consequence of (i) and (ii). $\square$

The alternative characterisation (2.2) might suggest that there is a relation between the pseudo numerical range $W_\Psi (T)$ and the closure $\overline{W(T)}\cap \Omega$ of the numerical range *W*(*T*) in $\Omega$. However, in general, there is no inclusion either way between them, see e.g. Example 3.2 where $W_\Psi (T)\not \subseteq \overline{W(T)}\cap \Omega$ and Example 2.9 where $\overline{W(T)} \cap \Omega \not \subseteq W_\Psi (T)$.

In fact, it was already noted in [31,  Prop. 2.9], for continuous functions of *bounded* operators and for the more general case of block numerical ranges, that, for $\uplambda \in \Omega$,

$$\begin{aligned} \uplambda \in \overline{W(T)} \implies 0 \in \overline{W(T(\uplambda ))}; \end{aligned}$$

the converse holds only under additional assumptions. More precisely, for families of bounded linear operators however, the following is known.

### Theorem 2.5

[31,  Prop. 2.9, Prop. 2.12, Thm. 2.14]

(i) If $\,T$ is a (norm-)continuous family of bounded linear operators, then
  $$\begin{aligned} \overline{W(T)} \cap \Omega \subseteq W_\Psi (T). \end{aligned}$$
(ii) If $\,T$ is a holomorphic family of bounded linear operators and there exist $k\in {\mathbb {N}}_0$ and $\mu \in \Omega$ with
  $$\begin{aligned} 0\notin \overline{W(T^{(k)}(\mu ))}, \end{aligned}$$
  then
  2.3 $$\begin{aligned} \sigma (T) \subseteq \overline{W(T)} \cap \Omega =W_\Psi (T). \end{aligned}$$

The following simple example from [31,  Ex. 2.11], which is easily adapted to the unbounded case, shows that condition (2.3) is essential for the equality $\overline{W(T)} \cap \Omega =W_\Psi (T)$ and for the spectral inclusion $\sigma (T) \subseteq \overline{W(T)} \cap \Omega$.

### Example 2.6

Let $f:\Omega \rightarrow {\mathbb {C}}$ be holomorphic, $f \not \equiv 0$, *A* a bounded or unbounded linear operator in ${\mathcal {H}}$ with $0\in \sigma (A)$, $0 \in \overline{W(A)}{\setminus } W(A)$ and consider

$$\begin{aligned} T(\uplambda ) := f(\uplambda ) A, \quad {\text {dom}}T(\uplambda ) {:}{=}{\text {dom}}A, \quad \uplambda \in \Omega . \end{aligned}$$

Then (2.3) is violated because, for any $k\in {\mathbb {N}}_0$ and $\mu \in \Omega$, we have $T^{(k)}(\mu ) = f^{(k)}(\mu ) A$ with ${\text {dom}}T^{(k)}(\uplambda ) = {\text {dom}}A$, $\uplambda \in \Omega$, and so $0\!\in \! \overline{W(T^{(k)}(\mu ))}$ since $0 \!\in \! \overline{W(A)}$. Further, it is easy to see that

$$\begin{aligned} \sigma (T)=\Omega , \quad W(T)= \overline{W(T)}\cap \Omega =\{ z \in \Omega : f(z)=0\} \ne \Omega , \quad W_\Psi (T)=\Omega . \end{aligned}$$

Thus neither $\overline{W(T)} \cap \Omega =W_\Psi (T)$ nor the spectral inclusion $\sigma (T) \subseteq \overline{W(T)}\cap \Omega$ hold, while $\sigma (T) = W_\Psi (T)$.

In the sequel we generalise Theorem 2.5 (i) and (ii) to families of unbounded operators and/or forms, including operator polynomials and sectorial families with constant form domain. In the remaining part of this section, we study the relation between $W_\Psi (T)$ and $\overline{W(T)}\cap \Omega$; results containing spectral enclosures may be found in Sect. 3.

### Proposition 2.7

Let *T* be an operator polynomial in ${\mathcal {H}}$ of degree $n\in {\mathbb {N}}$ with (possibly unbounded) coefficients $A_k:{\mathcal {H}}\supseteq {\text {dom}}A_k\rightarrow {\mathcal {H}}$, i.e.

$$\begin{aligned} T(\uplambda ){:}{=}\sum _{k=0}^n\uplambda ^k A_k, \quad {\text {dom}}T(\uplambda ){:}{=}\displaystyle \bigcap _{k=0}^n{\text {dom}}A_k, \quad \uplambda \in {\mathbb {C}}. \end{aligned}$$

If  $$0\notin \overline{W(A_n)}$$, then

$$\begin{aligned} W_\Psi (T)\subseteq \overline{W(T)}\cap \Omega , \end{aligned}$$

and analogously for form polynomials.

### Proof

Let $\uplambda _0\in W_{\Psi }(T)$. By Proposition 2.3, there is a sequence $\{f_m\}_m\subseteq {\text {dom}}T(\uplambda _0)$ with $\left\Vert f_m\right\Vert =1$, $m\in {\mathbb {N}}$, and $(T(\uplambda _0)f_m,f_m)\rightarrow 0$ for $m\rightarrow \infty$. Since $0\notin W(A_n)$ by assumption, the complex polynomial

$$\begin{aligned} p_m(\uplambda ){:}{=}(T(\uplambda )f_m,f_m)=\sum _{k=0}^n(A_kf_m,f_m)\uplambda ^k, \quad \uplambda \in {\mathbb {C}}, \end{aligned}$$

has degree *n* for each $m\in {\mathbb {N}}$. Let $\uplambda ^m_1,\dotsc ,\uplambda ^m_n\in {\mathbb {C}}$ denote its zeros. Then $\uplambda ^m_j\in W(T)$, $j=1,\dotsc ,n$, and $p_m$ admits the factorisation

$$\begin{aligned} p_m(\uplambda )=(A_nf_m,f_m)\prod _{j=1}^{n}(\uplambda -\uplambda ^m_j), \quad \uplambda \in {\mathbb {C}}, \quad m\in {\mathbb {N}}. \end{aligned}$$

Since $p_m(\uplambda _0)\rightarrow 0$ for $m\rightarrow \infty$ and $0\notin \overline{W(A_n)}$, there exists $j_0\in \{1,\dotsc ,n\}$ with $\uplambda ^m_{j_0}\rightarrow \uplambda _0$, $m\rightarrow \infty$, thus $\uplambda _0\in \overline{W(T)}$ and $\uplambda _0 \in W_\Psi (T) \subseteq \Omega$. $\square$

Next we generalise Theorem 2.5 (i) to families of sectorial forms with constant domain which satisfy a natural continuity assumption, see [17,  Thm. VI.3.6]. This assumption is met, in particular, by holomorphic form families of type (a) and associated operator families of type (B).

Recall that a family ${\mathbf {t}}$ of densely defined closed sectorial sesquilinear forms in ${\mathcal {H}}$ is called holomorphic of type (a) if its domain is constant and the mapping $\uplambda \mapsto {\mathbf {t}}(\uplambda )[f]$ is holomorphic for every $f\in {\mathcal {D}}_{\mathbf {t}}\!{:}{=}\!{\text {dom}}{\mathbf {t}}(\uplambda )$. The associated family *T* of m-sectorial operators is called holomorphic of type (B), see [17,  Sect. VII.4.2] and also [30]. Sufficient conditions on form families to be holomorphic of type (a) can be found in [17,  §VII.4].

### Theorem 2.8

Let ${\mathbf {t}}$ be a family of sectorial sesquilinear forms in ${\mathcal {H}}$ with constant domain ${\mathcal {D}}_{\mathbf {t}}{:}{=}{\text {dom}}{\mathbf {t}}(\uplambda )$, $\uplambda \in \Omega$. Assume that for each $\uplambda _0\in \Omega$, there exist *r*, $C>0$ and $w:B_r(\uplambda _0)\rightarrow [0,\infty )$, $\lim _{\uplambda \rightarrow \uplambda _0}w(\uplambda )=0$, such that

2.4 $$\begin{aligned} \left|{\mathbf {t}}(\uplambda _0)[f]-{\mathbf {t}}(\uplambda )[f]\right|\le w(\uplambda )\left( \left|{\text {Re}}{\mathbf {t}}(\uplambda _0)[f]\right|+C\left\Vert f\right\Vert ^2\right) \end{aligned}$$

for all $\uplambda \in B_r(\uplambda _0)$ and $f\in {\mathcal {D}}_{\mathbf {t}}$. Then

$$\begin{aligned} \overline{W({\mathbf {t}})}\cap \Omega \subseteq W_\Psi ({\mathbf {t}}). \end{aligned}$$

In particular, if ${\mathbf {t}}$ is a holomorphic form family of type (a) with associated holomorphic operator family *T* of type (B) in ${\mathcal {H}}$, then

2.5 $$\begin{aligned} \overline{W(T)}\cap \Omega \subseteq W_\Psi (T), \qquad \overline{W({\mathbf {t}})}\cap \Omega \subseteq W_\Psi ({\mathbf {t}}). \end{aligned}$$

### Proof

Let $\uplambda _0\in \overline{W({\mathbf {t}})}$. Then there exist $\{\uplambda _n\}_n\subseteq \Omega$ and $\{f_n\}_n\subseteq {\mathcal {D}}_{\mathbf {t}}$ with $\left\Vert f_n\right\Vert =1$, ${\mathbf {t}}(\uplambda _n)[f_n]=0$, $n\in {\mathbb {N}}$, and $\uplambda _n\rightarrow \uplambda _0$, $n\rightarrow \infty$. We show that ${\mathbf {t}}(\uplambda _0)[f_n]\!\rightarrow \!0$ for $n\!\rightarrow \!\infty$ which, in view of (2.2), implies $\uplambda _0\!\in \! W_\Psi ({\mathbf {t}})$. By (2.4),

$$\begin{aligned} \left|{\mathbf {t}}(\uplambda _0)[f_n]\right|=\left|{\mathbf {t}}(\uplambda _0)[f_n]-{\mathbf {t}}(\uplambda _n)[f_n]\right|\le w(\uplambda _n)\left( |{\text {Re}}{\mathbf {t}}(\uplambda _0)[f_n]|+C\right) , \quad n\in {\mathbb {N}}. \end{aligned}$$

Since $\left|{\text {Re}}{\mathbf {t}}(\uplambda _0)[f_n]\right|\le \left|{\mathbf {t}}(\uplambda _0)[f_n]\right|$ and $w(\uplambda _n)\rightarrow 0$, $n\rightarrow \infty$, we obtain that, for $n\in {\mathbb {N}}$ sufficiently large,

$$\begin{aligned} \left|{\mathbf {t}}(\uplambda _0)[f_n]\right|\le C\frac{w(\uplambda _n)}{1-w(\uplambda _n)}\longrightarrow 0, \quad n\rightarrow \infty . \end{aligned}$$

Now suppose that ${\mathbf {t}}$ and *T* are holomorphic families of type (a) and (B), respectively. We only need to show the second inclusion, the first one then follows from $W(T)\subseteq W({\mathbf {t}})$ and Corollary 2.4 (ii). The second inclusion follows from what we already proved since for holomorphic form families of type (a), after a possible shift ${\mathbf {t}}\!+\!c$ where $c\!>\!0$ is sufficiently large to ensure ${\text {Re}}{\mathbf {t}}(\uplambda _0)\!\ge \!1$, [17,  Eqn. VII.(4.7)] shows that assumption (2.4) is satisfied. $\square$

Theorem 2.5 (i) does not extend to analytic families of sectorial linear operators with non-constant form domains, as the following example inspired by [17,  Ex. VII.1.4] illustrates.

### Example 2.9

Let ${\mathcal {H}}=L^2(0,1)$. The family $T(\uplambda )$, $\uplambda \in {\mathbb {C}}$, given by

$$\begin{aligned} \begin{aligned} T(\uplambda )f&{:}{=}-f''-\uplambda f, \\ {\text {dom}}T(\uplambda )&{:}{=}\left\{ f\in H^2(0,1):f(0)=0, \, \uplambda f'(1)=f(1)\right\} , \end{aligned} \end{aligned}$$

is a holomorphic family of m-sectorial operators, but not holomorphic of type (B). Below we will show that

$$\begin{aligned} 0\in \overline{W(T)} \subseteq \overline{W_\Psi (T)}, \qquad 0\notin W_\Psi (T) ; \end{aligned}$$

note that, since $\Omega \!=\! {\mathbb {C}}$, this implies that the conclusion of Theorem 2.5 (i) does not hold and that $W_\Psi (T)$ is not closed in ${\mathbb {C}}$. Indeed, it is not difficult to check that the forms associated to $T(\uplambda )$, $\uplambda \in {\mathbb {C}}$,

$$\begin{aligned} {\mathbf {t}}(0) [f] = \Vert f'\Vert ^2, \quad {\mathbf {t}}(\uplambda ) [f] = \Vert f'\Vert ^2 - \uplambda \left\Vert f\right\Vert ^2 - \frac{1}{\uplambda }|f(1)|^2, \quad \uplambda \in \!{\mathbb {C}}\!{\setminus }\!\{0\}, \end{aligned}$$

are densely defined, closed and sectorial, but have $\uplambda$-depending domain ${\text {dom}}{\mathbf {t}}(0) \!=\! H_0^1(0,1)$ and ${\text {dom}}{\mathbf {t}}(\uplambda )\!=\! \left\{ f \in H^1(0,1):f(0)=0\right\}$ for $\uplambda \in \!{\mathbb {C}}\!{\setminus }\!\{0\}$. The holomorphy of the family follows from the holomorphy of the integral kernel, i.e. the Green’s function, of $(T(\uplambda )-\mu )^{-1}$, which, for $\uplambda \in {\mathbb {C}}$ and $\mu \in \rho (T(\uplambda ))\ne \emptyset$, is given by

$$\begin{aligned} G(x,y;\mu ,\uplambda )= \frac{\sin (\sqrt{\mu \!+\!\uplambda }x)(\sin (\sqrt{\mu \!+\!\uplambda }(1\!-\!y))\!-\!\uplambda \sqrt{\mu \!+\!\uplambda }\cos (\sqrt{\mu \!+\!\uplambda }(1\!-\!y)))}{\sqrt{\mu \!+\!\uplambda }(\sin \sqrt{\mu \!+\!\uplambda }-\uplambda \sqrt{\mu \!+\!\uplambda }\cos \sqrt{\mu \!+\!\uplambda } )} \end{aligned}$$

for $0\le x\le y\le 1$ and $G(x,y;\mu ,\uplambda )=G(y,x;\mu ,\uplambda )$ for $0\le y\le x\le 1$, cf. [17,  Ex. V.4.14, VII.1.5, VII.1.11] where the family $T(\uplambda ) + \uplambda$, $\uplambda \in {\mathbb {C}}$, was studied.

For fixed $\uplambda \in {\mathbb {C}}$, the spectrum of $T(\uplambda )$ is given by the singularities of the integral kernel $G(\cdot ,\cdot ;\mu ,\uplambda )$,

$$\begin{aligned} \begin{aligned} \sigma (T(\uplambda )) {\setminus } \{-\uplambda \}&\!=\!\sigma _{{\text {p}}}(T(\uplambda )) {\setminus } \{-\uplambda \} \!=\!\big \{\mu \!\in \!{\mathbb {C}}{\setminus } \{-\uplambda \}:\uplambda \sqrt{\mu \!+\!\uplambda }=\tan \sqrt{\mu \!+\!\uplambda }\big \}. \end{aligned} \end{aligned}$$

For $\uplambda \in (0,\infty )$ the operator $T(\uplambda )$ is self-adjoint and unbounded from above, and for $\uplambda \!\in \! (0,1)$ it has an eigenvalue $\mu _\uplambda \in \sigma _{{\text {p}}}(T(\uplambda )) \subseteq W(T(\uplambda ))$ of the form $\mu _\uplambda = -\uplambda - \kappa _\uplambda ^2 <0$ where $\kappa _\uplambda$ is the unique positive solution of $\tanh \kappa = \uplambda \kappa$. Thus $0 \in W(T(\uplambda ))$ for $\uplambda \in (0,1)$ due to the convexity of $W(T(\uplambda ))$, which proves $(0,1) \subseteq W(T) \subseteq W_\Psi (T)$ and thus $0\in \overline{W(T)}$ . On the other hand, $0\notin \overline{W(T(0))}=[\pi ^2,\infty )$ and so Proposition 2.3 implies $0\notin W_\Psi (T)$.

## Spectral Enclosure via Pseudo Numerical Range

In this section we derive spectral enclosures for families of unbounded linear operators $T(\uplambda )$, $\uplambda \in \Omega$, using the pseudo numerical range $W_\Psi (T)$. The latter is tailored to enclose the approximate point spectrum.

The spectrum and resolvent set of an operator family $T(\uplambda )$, $\uplambda \in \Omega$, respectively, are defined as

$$\begin{aligned} \sigma (T):=\left\{ \uplambda \in \Omega :0\in \sigma (T(\uplambda ))\right\} \subseteq \Omega , \quad \rho (T):=\Omega {\setminus } \sigma (T), \end{aligned}$$

and analogously for the various subsets of the spectrum. In addition to the approximate point spectrum

$$\begin{aligned} \sigma _{{\text {ap}}}(T) {:}{=}\left\{ \uplambda \in \Omega :\exists \, \{f_n\}_{n}\subseteq {\text {dom}}T(\uplambda ), \left\Vert f_n\right\Vert =1, T(\uplambda )f_n\rightarrow 0, n\rightarrow \infty \right\} , \end{aligned}$$

we introduce the $\varepsilon$-*approximate point spectrum*, see [22] for the operator case,

3.1 $$\begin{aligned} \sigma _{\mathrm{ap}, \varepsilon }(T) {:}{=}\left\{ \uplambda \in \Omega :\exists \, f\in {\text {dom}}T(\uplambda ), \,\left\Vert f\right\Vert =1, \, \left\Vert T(\uplambda )f\right\Vert <\varepsilon \right\} . \end{aligned}$$

The latter is a subset of the $\varepsilon$-pseudo spectrum

$$\begin{aligned} \sigma _\varepsilon (T) := \sigma _{\mathrm{ap}, \varepsilon }(T) \cup \sigma (T), \end{aligned}$$

which was defined for operator functions with unbounded closed values in [8,  Sect. 9.2, (9.9)], comp. also [7].

Clearly, for monic linear polynomials $T(\uplambda )= A \!-\! \uplambda I_{{\mathcal {H}}}$, $\uplambda \!\in \! {\mathbb {C}}$, these notions coincide with the spectrum, resolvent set, approximate point spectrum, $\varepsilon$-approximate point spectrum and $\varepsilon$-pseudo spectrum of the linear operator *A*.

### Proposition 3.1

For any operator family $T(\uplambda )$, $\uplambda \in \Omega$, and every $\varepsilon > 0$, we have $\sigma _{\mathrm{ap}, \varepsilon }(T) \subseteq W_\varepsilon (T)$,

$$\begin{aligned} \left\Vert T(\uplambda )^{-1}\right\Vert \le \frac{1}{\varepsilon }, \quad \uplambda \in \rho (T){\setminus } W_\varepsilon (T), \end{aligned}$$

and hence

$$\begin{aligned} \sigma _{{\text {ap}}}(T)\subseteq W_\Psi (T). \end{aligned}$$

If $\sigma (T(\uplambda ))\subseteq \overline{W(T(\uplambda ))}$ for all $\uplambda \in \Omega$, then

$$\begin{aligned} \sigma (T)\subseteq W_\Psi (T). \end{aligned}$$

### Proof

The claims follow easily from (3.1) and Definition 2.1 together with Cauchy-Schwarz’ inequality and (2.1) in Proposition 2.3. $\square$

The following simple examples illustrate some properties of the set $W_\Psi (T)$ versus $\overline{W(T)}\cap \Omega$, in particular, in view of spectral enclosures.

### Example 3.2

(i) Let $A\!>\!0$ be self-adjoint in ${\mathcal {H}}$ with $0\!\in \!\sigma (A)$. Then, for the non-holomorphic family $T(\uplambda )\!=\!A\!+\!\left|\sin \uplambda \right|$, $\uplambda \!\in \! \Omega \!:={\mathbb {C}}$, it is easy to see that
  $$\begin{aligned} W_\Psi (T)=\sigma (T)=\left\{ k\pi :k\in {\mathbb {Z}}\right\} \not \subseteq \overline{W(T)}\cap \Omega =\emptyset ; \end{aligned}$$
  notice that this implies $\overline{W_\Psi (T)}\cap \Omega \ne \overline{W(T)}\cap \Omega$, i.e. the closures of $W_\Psi (T)$ and *W*(*T*) in $\Omega$ do not coincide.
(ii) Let *A* be bounded in ${\mathcal {H}}$ with ${\text {Re}}W(A)>0$, $0\in \sigma (A)$ and $0\notin W(A)$. Consider the holomorphic family of bounded operators in ${\mathcal {H}}\oplus {\mathcal {H}}$
  $$\begin{aligned} T(\uplambda )=\left( \begin{array}{cc} \uplambda A &{} 0 \\ 0 &{} \uplambda {\text {Log}}(\uplambda +1) I_{{\mathcal {H}}} \end{array}\right) , \quad \uplambda \in \Omega := {\mathbb {C}}{\setminus }(-\infty ,-1]; \end{aligned}$$
  here ${\text {Log}}:{\mathbb {C}}{\setminus }(-\infty ,0]\rightarrow \left\{ z\in {\mathbb {C}}:{\text {Im}}z\in (-\pi ,\pi ]\right\}$ denotes the principal value of the complex logarithm. This family does not satisfy condition (2.3) in Theorem 2.5 since $0 \in \overline{W(A)}$ by assumption. It is not difficult to show that
  $$\begin{aligned} W_\Psi (T)=\sigma (T)= {\mathbb {C}}{\setminus }(-\infty ,-1] \not \subseteq \overline{W(T)} \cap \Omega \subseteq \overline{B_1(-1)} {\setminus } [-2,-1]. \end{aligned}$$
  In fact, the claims for $W_\Psi (T)$ are obvious. If $\uplambda \!\in \! W(T)$, then $\uplambda \!\in \!{\mathbb {C}}\!{\setminus }\!(-\infty ,-1]$ and there exists $x\!=\!(f,g)^{\mathrm{t}} \!\in \! {\mathcal {H}}\oplus {\mathcal {H}}$, $(f,g)^{\mathrm{t}}\ne (0,0)^{\mathrm{t}}$, with
  $$\begin{aligned} \big ( T(\uplambda ) x,x \big ) = \uplambda \big ( (Af,f) + (\ln |\uplambda +1| + \mathrm{i} \arg (\uplambda +1) ) (g,g)\big ) = 0 \end{aligned}$$
  or, equivalently, noting that $\uplambda \ne 0$ implies $g\ne 0$ as $0 \notin W(A)$,
  $$\begin{aligned} \uplambda =0 \ \vee \ \Big ( |\uplambda \!+\!1| \!=\! \exp \Big (\!-\!\frac{{\text {Re}}(Af,f)}{(g,g)}\Big ) \wedge \arg (\uplambda \!+\!1) \!=\! - \frac{{\text {Im}}(Af,f)}{(g,g)} \Big ). \end{aligned}$$
  Hence, since $${\text {Re}}W(A)>0$$,
  $$\begin{aligned} W(T) {\setminus } \{0\} \! \subseteq \! \big \{ z\!\in \! {\mathbb {C}}{\setminus }(-\infty ,-1] \,:\, |z\!+\!1| \in (0,1)\big \} \subseteq B_1(-1) {\setminus } (-2,-1]. \end{aligned}$$
  Moreover, for arbitrary $h\in {\mathcal {H}}$, $h\ne 0$,
  $$\begin{aligned} \left( T\left( \exp {\Big (\!-\!\frac{(Ah,h)}{(h,h)}\Big )} -1\right) \left( {\begin{array}{c}h\\ h\end{array}}\right) , \left( {\begin{array}{c}h\\ h\end{array}}\right) \right) =0. \end{aligned}$$
  This shows that $\left\{ \exp (-z) - 1:z \in W(A)\right\} \subseteq W(T)$ and since $\exp$ is entire and non-constant, $W(A)^\circ \ne \emptyset$ implies that $W(T)^\circ \ne \emptyset$ by the open mapping theorem for holomorphic functions. So in this case $W_\Psi (T)^\circ \ne W(T)^\circ$ and both are non-empty.
  $$\begin{aligned} W_\Psi (T)^\circ \!=\!{\mathbb {C}}{\setminus }(-\infty ,-1], \quad \emptyset \ne W(T)^\circ \subseteq B_1(-1) {\setminus } (-2,-1]. \end{aligned}$$

In the following, we generalise the spectral enclosure for bounded holomorphic families in Theorem 2.5 (ii) to holomorphic form families ${\mathbf {t}}$ of type (a) and associated operator families of type (B), i.e. ${\mathbf {t}}(\uplambda )$ is sectorial with vertex $\gamma (\uplambda )\!\in \!{\mathbb {R}}$, semi-angle $\vartheta (\uplambda )\!\in \! [0,\frac{\pi }{2})$ and $\uplambda$-independent domain ${\text {dom}}{\mathbf {t}}(\uplambda )\!=\! {\mathcal {D}}_{\mathbf {t}}$. Here, for $k \in {\mathbb {N}}_0$, we denote the *k*-th derivative of ${\mathbf {t}}$ by

$$\begin{aligned} {\mathbf {t}}^{(k)}(\uplambda )[f] {:}{=}({\mathbf {t}}(\cdot )[f])^{(k)}(\uplambda ), \quad f \in {\text {dom}}{\mathbf {t}}^{(k)}(\uplambda ) {:}{=}{\mathcal {D}}_{\mathbf {t}}= {\text {dom}}{\mathbf {t}}(\uplambda ), \quad \uplambda \in \Omega ; \end{aligned}$$

note that ${\mathbf {t}}^{(k)}(\uplambda )$ need not be closable or sectorial if $k>0$.

### Theorem 3.3

Let ${\mathbf {t}}$ be a holomorphic form family of type (a) with associated holomorphic operator family *T* of type (B) in ${\mathcal {H}}$. If there exist $k\in {\mathbb {N}}_0$, $\mu \in \Omega$ and a core ${\mathcal {D}}$ of ${\mathbf {t}}(\mu )$ with

3.2 $$\begin{aligned} 0 \notin \overline{W\big ({\mathbf {t}}^{(k)}(\mu )\big |_{\mathcal {D}}\big )}, \end{aligned}$$

then

$$\begin{aligned} \sigma (T) \subseteq W_\Psi ({\mathbf {t}})=\overline{W({\mathbf {t}})} \cap \Omega . \end{aligned}$$

If, in addition, the operator family *T* has constant domain, then

$$\begin{aligned} \sigma (T) \!\subseteq \, W_\Psi (T)=\overline{W(T)}\cap \Omega . \end{aligned}$$

### Remark 3.4

(i) Since ${\mathbf {t}}(\uplambda )$ is densely defined, closed and sectorial for all $\uplambda \!\in \! \Omega$, condition (3.2) for $k=0$ has the two equivalent forms
  $$\begin{aligned} 0 \notin \overline{W\big ({\mathbf {t}}(\mu )\big |_{\mathcal {D}}\big )} \ \iff \ 0 \notin \overline{W(T(\mu ))}; \end{aligned}$$
  hence, by Proposition 2.3 a sufficient condition for (3.2) is
  $$\begin{aligned} W_\Psi (T)\ne \Omega . \end{aligned}$$
(ii) For operator polynomials *T*, which are holomorphic and have constant domain by definition, see Proposition 2.7, no sectoriality assumption is needed for the enclosure
  $$\begin{aligned} \sigma _{{\text {ap}}}(T) \subseteq W_\Psi (T) \subseteq \overline{W(T)} \cap \Omega . \end{aligned}$$
  By Propositions 2.7 and 3.1, the above holds under the mere assumption that $0 \notin \overline{W(A_n)}$ where $A_n$ is the leading coefficient of *T*; note that then (3.2) holds with $k=n$ and arbitrary $\mu \in {\mathbb {C}}$. This generalises the classical result [20,  Thm. 26.7] for bounded operator polynomials; see also [31,  Prop. 3.3] for the block numerical range.
(iii) In general, neither the assumption on holomorphy nor condition (3.2) in Theorem 3.3 can be omitted, see Examples 2.6 and 3.2.

### Proof of Theorem 3.3

First we show that if condition (3.2) holds for some core ${\mathcal {D}}$ of ${\mathbf {t}}(\mu )$, it also holds for ${\mathcal {D}}$ replaced by ${\mathcal {D}}_{\mathbf {t}}= {\text {dom}}{\mathbf {t}}(\uplambda )$, $\uplambda \in \Omega$. For $k\!=\!0$, this follows from the properties of a core, see [17,  Thm. VI.1.18]. For $k>0$, without loss of generality, we may assume that ${\text {Re}}{\mathbf {t}}(\mu ) \ge 1$. From the proof of [17,  Eqn. VII.(4.7)], it is easy to see that the second inequality therein holds for ${\mathbf {t}}^{(k)}$, i.e. there exists a constant $C_\mu >0$ such that

3.3 $$\begin{aligned} \big |{\mathbf {t}}^{(k)}(\mu )[f,g]\big | \le C_\mu \left|{\mathbf {t}}(\mu ) [f]\right|^\frac{1}{2}\left|{\mathbf {t}}(\mu ) [g]\right|^\frac{1}{2}, \quad f,g \in {\mathcal {D}}_{\mathbf {t}}. \end{aligned}$$

To prove the claim stated at the beginning assume, to the contrary, that $0 \in \overline{W({\mathbf {t}}^{(k)}(\mu ))}$, i.e. that there exists a sequence $\{f_n\}_n \subseteq {\mathcal {D}}_{\mathbf {t}}$, $\left\Vert f_n\right\Vert =1$, $n\in {\mathbb {N}}$, with ${\mathbf {t}}^{(k)}(\mu )[f_n] \!\rightarrow \! 0$ as $n\!\rightarrow \!\infty$. By the core property of ${\mathcal {D}}$ for ${\mathbf {t}}[\mu ]$ and by [17,  Thm. VI.1.12], for fixed $n\in {\mathbb {N}}$, there exists $\{f_{n,m}\}_m\subseteq {\mathcal {D}}$ with

3.4 $$\begin{aligned} f_{n,m} \!\rightarrow \! f_n, \quad {\mathbf {t}}(\mu )[f_{n,m}\!-\!f_n]\!\rightarrow \! 0, \quad {\mathbf {t}}(\mu )[f_{n,m}] \!\rightarrow \! {\mathbf {t}}(\mu )[f_n], \quad m\!\rightarrow \! \infty . \end{aligned}$$

Applying (3.3), we can estimate

$$\begin{aligned} \begin{aligned} \big |{\mathbf {t}}^{\!(k)}(\mu ) [f_{n,m}] \!-\! {\mathbf {t}}^{\!(k)}(\mu ) [f_n] \big |&\!\le \! \big |{\mathbf {t}}^{\!(k)}(\mu ) [f_{n,m}, f_{n,m}\!-\!f_n]\big | \!+\! \big |{\mathbf {t}}^{\!(k)}(\mu ) [f_n \!-\! f_{n,m}, f_n]\big |\\&\!\le \! C_\mu \left|{\mathbf {t}}(\mu ) [f_{n,m} \!-\! f_n]\right|^\frac{1}{2}\!\big (\left|{\mathbf {t}}(\mu ) [f_{n,m}]\right|^\frac{1}{2} \!\!+\! \left|{\mathbf {t}}(\mu ) [f_n]\right|^\frac{1}{2}\!\big ). \end{aligned} \end{aligned}$$

Since $\left\Vert f_n\right\Vert \!=\!1$, $n\!\in \!{\mathbb {N}}$, it follows from (3.4) and the above inequality that there exists $m_n\ge n$ such that

$$\begin{aligned} \left\Vert f_{n,m_n}\right\Vert \ge \frac{1}{2}, \quad \left|{\mathbf {t}}^{(k)}(\mu )[f_{n,m_n}]\right|<\left|{\mathbf {t}}^{(k)}(\mu )[f_n]\right|+\frac{1}{n}. \end{aligned}$$

In view of ${\mathbf {t}}^{(k)}(\mu ) [f_n] \rightarrow 0$, $n \rightarrow \infty$, this implies the required claim

$$\begin{aligned} 0 \in \overline{W\big ({\mathbf {t}}^{(k)}(\mu ) \big |_{\mathcal {D}}\big )}. \end{aligned}$$

This completes the proof that (3.2) holds with ${\mathcal {D}}_{\mathbf {t}}$ instead of ${\mathcal {D}}$.

By Corollary 2.4 (ii), we have $W_\Psi ({\mathbf {t}})=W_\Psi (T)\subseteq \Omega$. Thus, due to (2.5), for the claimed equalities between pseudo numerical and numerical ranges it is sufficient to show $W_\Psi ({\mathbf {t}})\subseteq \overline{W({\mathbf {t}})}$ and $W_\Psi ({\mathbf {t}})\subseteq \overline{W(T)}$, respectively.

Let $\uplambda _0\in W_\Psi ({\mathbf {t}})=W_\Psi (T)$. Then $0\in \overline{W(T(\uplambda _0))}$ by Proposition 2.3 and hence there exists $\{f_n\}_n\!\subseteq \!{\text {dom}}T(\uplambda _0)\!\subseteq \!{\mathcal {D}}_{\mathbf {t}}$ with $\left\Vert f_n\right\Vert \!=\!1$, $n\in {\mathbb {N}}$, such that

3.5 $$\begin{aligned} \left( T(\uplambda _0)f_n,f_n\right) ={\mathbf {t}}(\uplambda _0)[f_n]\rightarrow 0, \quad n\rightarrow \infty . \end{aligned}$$

Define a sequence of holomorphic functions

$$\begin{aligned} \varphi _n(\uplambda ){:}{=}{\mathbf {t}}(\uplambda ) [f_n], \quad \uplambda \in \Omega , \quad n\in {\mathbb {N}}. \end{aligned}$$

Let $K\subseteq \Omega$ be an arbitrary compact subset and let $c>0$ be such that ${\text {Re}}({\mathbf {t}}+c) (\uplambda _0) \ge 1$. By [17,  Eqn. VII.(4.7)], there exists $b_K>0$ with

$$\begin{aligned} |({\mathbf {t}}+c)(\uplambda )[f]|\le b_K |({\mathbf {t}}+c)(\uplambda _0)[f]|, \quad \uplambda \in K, \ f \in {\mathcal {D}}_{\mathbf {t}}. \end{aligned}$$

Using this, $\left\Vert f_n\right\Vert =1$ and (3.5), we find that, for all $\uplambda \in K$,

$$\begin{aligned} \left|\varphi _n(\uplambda )\right|\le b_{K} \left|({\mathbf {t}}+c)(\uplambda _0)[f_n]\right| +c\le b_{K} \sup _{n\in {\mathbb {N}}} \left|{\mathbf {t}}(\uplambda _0)[f_n]\right| +(b_K+1)c<\infty . \end{aligned}$$

Consequently, $\{\varphi _n\}_n$ is uniformly bounded on compact subsets of $\Omega$. By Montel’s Theorem, see e.g. [5,  §VII.2], there exists a subsequence $\{\varphi _{n_j}\}_j\subseteq \{\varphi _n\}_n$ that converges locally uniformly to a holomorphic function $\varphi$. Now assumption (3.2) with ${\mathcal {D}}_{\mathbf {t}}$, which we proved to hold in the first step, implies

$$\begin{aligned} \varphi ^{(k)}(\mu )=\frac{{\mathrm{d}}^k}{\mathrm{d}\!\uplambda ^k}\lim _{j\rightarrow \infty }\varphi _{n_j}(\uplambda )\bigg |_{\uplambda =\mu }=\lim _{j\rightarrow \infty }\varphi _{n_j}^{(k)}(\mu ) = \lim _{j\rightarrow \infty } {\mathbf {t}}^{(k)}(\mu ) [f_{n_j}] \ne 0 \end{aligned}$$

and thus $\varphi \not \equiv 0$. By (3.5), we further conclude that $\varphi (\uplambda _0)=0$. Then, by Hurwitz’ Theorem, see e.g. [5,  §VII.2], there exists a sequence $\{\uplambda _j\}_j\subseteq \Omega$ with $\uplambda _j\rightarrow \uplambda _0$ for $j\rightarrow \infty$ and

$$\begin{aligned} 0=\varphi _{n_j}(\uplambda _j)={\mathbf {t}}(\uplambda _j)[f_{n_j}], \quad j\in {\mathbb {N}}. \end{aligned}$$

Hence, $\uplambda _j\in W({\mathbf {t}})$ for all $j\in {\mathbb {N}}$ and so $\uplambda _0\in \overline{W({\mathbf {t}})}\cap \Omega$, as required.

Now assume that the operator family *T* has constant domain. Then, in the above construction, we have $f_{n_j} \in {\text {dom}}T(\uplambda _0) = {\text {dom}}T(\uplambda _j)$ for every $j\in {\mathbb {N}}$. It follows that $\uplambda _j\in W(T)$, $j\in {\mathbb {N}}$, and thus $\uplambda _0\in \overline{W(T)}\cap \Omega$.

The enclosures of the spectrum follow from Proposition 3.1 and from the fact that $\sigma (T(\uplambda )) \subseteq \overline{W(T(\uplambda ))}$ since $T(\uplambda )$ is m-sectorial for all $\uplambda \in \Omega$. $\square$

As forms are the natural objects regarding numerical ranges, it is not surprising that the inclusion $W_\Psi (T)\subseteq \overline{W(T)}\cap \Omega$ in Theorem 3.3 might cease to hold for more general analytic operator families where the connection to a family of forms is lost. Nevertheless, using an analogous idea as in the proof of Theorem 3.3, one can prove the corresponding inclusion for the approximate spectrum.

Recall that an operator family *T* in ${\mathcal {H}}$ is called holomorphic of type (A) if it consists of closed operators with constant domain and for each $f\in {\mathcal {D}}_T{:}{=}{\text {dom}}T(\uplambda )$, the mapping $\uplambda \mapsto T(\uplambda )f$ is holomorphic on $\Omega$. Here, for $k \in {\mathbb {N}}_0$, the *k*-th derivative of *T* is defined as

$$\begin{aligned} T^{(k)}(\uplambda )f {:}{=}(T(\cdot ) f)^{(k)}(\uplambda ), \quad f \in {\text {dom}}T^{(k)} (\uplambda ) {:}{=}{\mathcal {D}}_T, \quad \uplambda \in \Omega . \end{aligned}$$

### Theorem 3.5

Let *T* be a holomorphic family of type (A) in ${\mathcal {H}}$. If there exist $k\in {\mathbb {N}}_0$, $\mu \in \Omega$ and a core ${\mathcal {D}}$ of $T(\mu )$ with

3.6 $$\begin{aligned} 0 \notin \overline{W\big (T^{(k)}(\mu )\big |_{\mathcal {D}}\big )}, \end{aligned}$$

then

$$\begin{aligned} \sigma _{{\text {ap}}}(T)\subseteq \overline{W(T)} \cap \Omega . \end{aligned}$$

### Proof

In the same way as in the proof of Theorem 3.3, using the analogue of [17,  Eqn. VII.(2.3)] for the *k*-th derivative of *T* and Cauchy-Schwarz’ inequality, one shows that (3.6) holds with ${\mathcal {D}}_T \!=\! {\text {dom}}T(\uplambda )$, $\uplambda \!\in \!\Omega$, instead of ${\mathcal {D}}$.

We proceed similarly as in the proof of Theorem 3.3. Let $\uplambda _0\in \sigma _{{\text {ap}}}(T)$. There exists a sequence $\{f_n\}_n\subseteq {\mathcal {D}}_T$ with $\left\Vert f_n\right\Vert =1$, $n\in {\mathbb {N}}$, and $T(\uplambda _0)f_n\rightarrow 0$ as $n\rightarrow \infty$. Define a sequence of holomorphic functions

$$\begin{aligned} \varphi _n(\uplambda ){:}{=}\left( T(\uplambda )f_n,f_n\right) , \quad \uplambda \in \Omega , \quad n\in {\mathbb {N}}. \end{aligned}$$

Analogously to the proof of Theorem 3.3, one uses Cauchy-Schwarz’ inequality, equation [17,  Eqn. VII.(2.2)], $\lim _{n\rightarrow \infty }T(\uplambda _0)f_n=0$ and (3.6) with ${\mathcal {D}}_T$ in order to show uniform boundedness of $\{\varphi _n\}_n$ on compacta, extract a locally uniformly converging subsequence with limit $\varphi \not \equiv 0$ and infer $\varphi (\uplambda _0)=0$. One then obtains $\uplambda _0\in \overline{W(T)}\cap \Omega$ in the same way as in Theorem 3.3. $\square$

### Remark 3.6

Theorems 3.3 and 3.5 generalise the classical result [20,  Thm. III. 26.6] for bounded holomorphic families (which follows from Theorem 2.5 (ii)).

Like for the numerical range of unbounded operators, cf. [17,  Sct. V.3.2], additional conditions are needed for enclosing not only the approximate point spectrum, but the entire spectrum $\sigma (T)$ in $W_\Psi (T)$.

### Remark 3.7

Let *T* be a family of closed operators in ${\mathcal {H}}$ and let *T* be continuous in the generalised sense. If $\sigma _{{\text {ap}}}(T)\subseteq \Theta \subseteq \Omega$ and all connected components of $\Omega {\setminus }\Theta$ contain a point in the resolvent set of *T*, then $\sigma (T)\subseteq \Theta$. In particular, if all connected components of $\Omega {\setminus } W_\Psi (T)$ have non-empty intersection with $\rho (T)$, then

$$\begin{aligned} \sigma (T)\subseteq W_\Psi (T). \end{aligned}$$

This follows from the fact that the index of $T(\uplambda )$ is locally constant on the set of regular points, see [17,  Thm. IV.5.17].

## Pseudo Block Numerical Ranges of Operator Matrix Functions and Spectral Enclosures

In this section we introduce the pseudo block numerical range of $n\times n$ operator matrix functions for which the entries may have unbounded operator values. While we study its basic properties for $n\ge 2$, we study the most important case $n=2$ in greater detail.

We suppose that with respect to a fixed decomposition ${\mathcal {H}}={\mathcal {H}}_1\oplus \cdots \oplus {\mathcal {H}}_n$ with $n\in {\mathbb {N}}$, a family ${\mathcal {L}}=\left\{ {\mathcal {L}}(\uplambda ):\uplambda \in \Omega \right\}$ of densely defined linear operators in ${\mathcal {H}}$ admits a matrix representation

$$\begin{aligned} {\mathcal {L}}(\uplambda )=\left( L_{ij} (\uplambda ) \right) _{i,j=1}^n :{\mathcal {H}}\supseteq {\text {dom}}{\mathcal {L}}(\uplambda )\rightarrow {\mathcal {H}}; \end{aligned}$$

here $L_{ij}$ are families of densely defined and closable linear operators from ${\mathcal {H}}_j$ to ${\mathcal {H}}_i$, *i*, $j=1,\dots , n$, and ${\text {dom}}{\mathcal {L}}(\uplambda )={\mathcal {D}}_1(\uplambda )\oplus \cdots \oplus {\mathcal {D}}_n(\uplambda )$,

$$\begin{aligned} {\mathcal {D}}_j(\uplambda ){:}{=}\bigcap _{i=1}^n {\text {dom}}L_{ij}(\uplambda ), \quad j=1,\dots ,n. \end{aligned}$$

The following definition generalises, and unites, several earlier concepts: the block numerical range of $n\times n$ operator matrix families whose entries have bounded linear operator values, see [23], the block numerical range of unbounded $n \times n$ operator matrices, see [24], and in the special case $n\!=\!2$, the quadratic numerical range for bounded analytic operator matrix families and unbounded operator matrices, see [28] and [19, 27], respectively. Further, we introduce the new concept of pseudo block numerical range.

### Definition 4.1

(i) We define the *block numerical range* of ${\mathcal {L}}$ (with respect to the decomposition ${\mathcal {H}}={\mathcal {H}}_1\oplus \cdots \oplus {\mathcal {H}}_n$) as
  $$\begin{aligned} W^{n}({\mathcal {L}}){:}{=}\{\uplambda \in \Omega : \exists \, f\in \!{\text {dom}}{\mathcal {L}}(\uplambda )\cap {{\mathcal {S}}}^n \ 0 \!\in \! \sigma ({\mathcal {L}}(\uplambda )_f)\} \end{aligned}$$
  where ${{\mathcal {S}}}^n{:}{=}\{ f\!=\!(f_i)_{i=1}^n \!\in \! {\mathcal {H}}: \left\Vert f_i\right\Vert \!=\!1, i\!=\!1,\dots ,n\}$ and, for $f\!=\!(f_i)_{i=1}^n\!\in \!{\text {dom}}{\mathcal {L}}(\uplambda )\cap {{\mathcal {S}}}^n$ with $\uplambda \!\in \! \Omega$,
  $$\begin{aligned} {\mathcal {L}}(\uplambda )_{f}{:}{=}\left( {\mathcal {L}}_{ij}(\uplambda ) f_j, f_i \right) \in {\mathbb {C}}^{n\times n}. \end{aligned}$$
(ii) We introduce the *pseudo block numerical range* of ${\mathcal {L}}$ as
  $$\begin{aligned} W^n_\Psi ({\mathcal {L}}){:}{=}\bigcap _{\varepsilon>0}W_\varepsilon ^n({\mathcal {L}}), \qquad W_\varepsilon ^n({\mathcal {L}}){:}{=}\bigcup _{{\mathcal {B}}\in L({\mathcal {H}}), \left\Vert {\mathcal {B}}\right\Vert <\varepsilon } W^n({\mathcal {L}}+{\mathcal {B}}), \quad \varepsilon >0. \end{aligned}$$

Note that, indeed, if ${\mathcal {L}}(\uplambda )\!=\!{\mathcal {A}}\!-\!\uplambda I_{\mathcal {H}}$, $\uplambda \!\in \! {\mathbb {C}}$, with an (unbounded) operator matrix ${\mathcal {A}}$ in ${\mathcal {H}}$, then ${\text {dom}}{\mathcal {L}}(\uplambda )\!=\!{\text {dom}}{\mathcal {A}}$ is constant for $\uplambda \!\in \!{\mathbb {C}}$ and $W^n({\mathcal {L}})$ coincides with the block numerical range $W^n({\mathcal {A}})$ first introduced in [24] and, for $n\!=\!2$, in [27]. While the pseudo numerical range also satisfies $W_\Psi ({\mathcal {L}})\!=\!\overline{W({\mathcal {L}})} = \overline{W({\mathcal {A}})}$ this is no longer true for the pseudo block numerical range when $n>1$; in fact, Example 4.5 below shows that $W_\Psi ^2({\mathcal {L}})\ne \overline{W^2({\mathcal {L}})} = \overline{W^{2}({\mathcal {A}})}$ is possible.

### Remark 4.2

It is not difficult to see that, for the block numerical range and the pseudo block numerical range of general operator matrix families,

4.1 $$\begin{aligned} \uplambda \in W^n({\mathcal {L}}) \iff 0\in W^n({\mathcal {L}}(\uplambda )) \end{aligned}$$

and $W^n({\mathcal {L}})\!\subseteq \! W^n_\Psi ({\mathcal {L}})$. If ${\text {dom}}{\mathcal {L}}(\uplambda )\!=:\!{\mathcal {D}}_{\mathcal {L}}$, $\uplambda \!\in \!\Omega$, is constant, we can also write

$$\begin{aligned} W^{n}({\mathcal {L}}){:}{=}\bigcup _{f\in {\mathcal {D}}_{\mathcal {L}}\cap {{\mathcal {S}}}^n} \sigma \big ( {\mathcal {L}}_{f} \big ). \end{aligned}$$

There are several other possible ways to define the pseudo block numerical range. In the following we show that, in general, they inevitably fail to contain the approximate point spectrum of an operator matrix family.

### Definition 4.3

Define

$$\begin{aligned} W_{\Psi ,0}^{n}({\mathcal {L}})\!{:}{=}\!\left\{ \uplambda \!\in \!\Omega :0\in \overline{W^{n}({\mathcal {L}}(\uplambda ))}\right\} , \quad W_{\Psi ,i}^{n}({\mathcal {L}})\!{:}{=}\!\bigcap _{\varepsilon >0}W_{\varepsilon ,i}^{n}({\mathcal {L}}), \ i\!=\!1,2, \end{aligned}$$

where, for $$\varepsilon >0$$,

$$\begin{aligned} \begin{aligned} W_{\varepsilon ,1}^{n}({\mathcal {L}})&\!{:}{=}\! \left\{ \uplambda \in \Omega :\exists \, f\in {\text {dom}}{\mathcal {L}}(\uplambda ) \cap {{\mathcal {S}}}^n, \left|\det ({\mathcal {L}}(\uplambda )_{f})\right|<\varepsilon \right\} \!, \\ W_{\varepsilon ,2}^{n}({\mathcal {L}})&\!{:}{=}\! \!\!\bigcup _{B_i\in L({\mathcal {H}}_i),\left\Vert B_i\right\Vert <\varepsilon } \!\!W^{n}\big ({\mathcal {L}}+{\text {diag}}(B_1,\dots ,B_n)\big ). \end{aligned} \end{aligned}$$

While for the pseudo numerical range, analogous concepts as in Definition 4.3 coincide by Proposition 2.3, this is not true for the pseudo block numerical range. Here, in general, we only have the following inclusions.

### Proposition 4.4

The pseudo block numerical range $W^{n}_\Psi ({\mathcal {L}})$ satisfies

4.2 $$\begin{aligned} W^n({\mathcal {L}}) \subseteq W^{n}_{\Psi ,1}({\mathcal {L}})\subseteq W_{\Psi ,0}^{n}({\mathcal {L}})\subseteq W^{n}_{\Psi ,2}({\mathcal {L}})\subseteq W^{n}_\Psi ({\mathcal {L}}). \end{aligned}$$

### Proof

We consider the case $n=2$; the proofs for $n>2$ are analogous. The leftmost and rightmost inclusions are trivial by definition. For the remaining inclusions, it is sufficient to show that, for every $\varepsilon >0$,

4.3 $$\begin{aligned} W^2_{\varepsilon ,1}({\mathcal {L}}) \subseteq \left\{ \uplambda \in \Omega :0\in {\text {B}}_{\sqrt{\varepsilon }}(W^2({\mathcal {L}}(\uplambda )))\right\} \subseteq W^2_{\sqrt{\varepsilon },2}({\mathcal {L}}). \end{aligned}$$

Then the respective claims follow by taking the intersection over all $\varepsilon >0$.

Let $\varepsilon >0$ and $\uplambda \in W_{\varepsilon ,1}^2({\mathcal {L}})$. Then there exists $f\in {\text {dom}}{\mathcal {L}}(\uplambda ) \cap {{\mathcal {S}}}^2$ with

$$\begin{aligned} \sigma ({\mathcal {L}}(\uplambda )_{f})=\{\uplambda _1,\uplambda _2\}\subseteq W^2({\mathcal {L}}(\uplambda )), \qquad \left|\uplambda _1\right|\left|\uplambda _2\right|=\left|\det {\mathcal {L}}(\uplambda )_{f}\right|<\varepsilon . \end{aligned}$$

Now the first inclusion in (4.3) follows from

$$\begin{aligned} {\text {dist}}(0,W^2({\mathcal {L}}(\uplambda )))\le \min \{\left|\uplambda _1\right|,\left|\uplambda _2\right|\}< \sqrt{\varepsilon }. \end{aligned}$$

For the second inclusion, let $\uplambda \!\in \!\Omega$ with ${\text {dist}}(0,W^2({\mathcal {L}}(\uplambda )))\!<\!\!\sqrt{\varepsilon }$, i.e. there exists $\mu \!\in \!{\mathbb {C}}$, $\left|\mu \right|\!<\!\!\sqrt{\varepsilon }$, with $\mu \!\in \! W^2({\mathcal {L}}(\uplambda ))$ or, equivalently, $0\!\in \! W^2({\mathcal {L}}(\uplambda )\!-\!\mu {\mathcal {I}}_{\mathcal {H}})$. By (4.1), the latter is in turn equivalent to

$$\begin{aligned} \uplambda \in W^2({\mathcal {L}}-\mu {\mathcal {I}}_{{\mathcal {H}}})\subseteq W^2_{\sqrt{\varepsilon },2}({\mathcal {L}}). \end{aligned}$$

$$\square $$

Clearly, in the simplest case ${\mathcal {L}}(\uplambda )={\mathcal {A}}-\uplambda I_{\mathcal {H}}$, $\uplambda \in {\mathbb {C}}$, with an $n\times n$ operator matrix ${\mathcal {A}}$ in ${\mathcal {H}}$ we have

$$\begin{aligned} W_{\Psi ,0}^{n}({\mathcal {L}})=\overline{W^{n}({\mathcal {L}})} =\overline{W^{n}({\mathcal {A}})}; \end{aligned}$$

this shows that $W_{\Psi ,0}^n({\mathcal {L}})$ fails to enclose the spectrum of ${\mathcal {L}}$ whenever $\overline{W^n({\mathcal {A}})}$ does.

The following example shows that, already in this simple case, in fact *none* of the subsets $W^n_{\Psi ,1}({\mathcal {L}})\subseteq W_{\Psi ,0}^n({\mathcal {L}})\subseteq W^n_{\Psi ,2}({\mathcal {L}})$ of the pseudo block numerical range $W^n_\Psi ({\mathcal {L}})$, see (4.2), is large enough to contain the approximate point spectrum $\sigma _{\mathrm{ap}}({\mathcal {L}})$.

### Example 4.5

Let ${\mathcal {H}}\!=\!\ell ^2({\mathbb {N}})\oplus \ell ^2({\mathbb {N}})$ and ${\mathcal {L}}(\uplambda )\!=\!{\mathcal {A}}-\uplambda I_{\mathcal {H}}$, $\uplambda \in {\mathbb {C}}$, with

$$\begin{aligned} {\mathcal {A}}{:}{=}\!\left( \begin{array}{cc} \!0 &{}\quad {\text {diag}}(m^2\!-\!1:m\!\in \!{\mathbb {N}})\!\! \\ \!0 &{}\quad 0\!\! \end{array}\right) , \ \ {\text {dom}}{\mathcal {A}}{:}{=}\ell ^2({\mathbb {N}})\,\oplus \,{\text {dom}}{\text {diag}}(m^2\!-\!1:m\!\in \!{\mathbb {N}}), \end{aligned}$$

where ${\text {diag}}(m^2-1:m\!\in \!{\mathbb {N}})$ is the unbounded maximal multiplication operator in $\ell ^2({\mathbb {N}})$ with domain

$$\begin{aligned}&{\text {dom}}{\text {diag}}(m^2\!-\!1:m\!\in \!{\mathbb {N}}) := \big \{\{x_m\}_m \in \ell ^2({\mathbb {N}}): \{(m^2\!-\!1)x_m\}_m \in \ell ^2({\mathbb {N}}) \big \}. \end{aligned}$$

Clearly, $W^2({\mathcal {L}})=W^2({\mathcal {A}})=\{0\}$. We will now show that

$$\begin{aligned} \{0\} \!= W_{\Psi ,1}^2({\mathcal {L}})\!=\!W_{\Psi ,0}^2({\mathcal {L}})\!=\!W_{\Psi ,2}^2({\mathcal {L}}) \ne W^2_\Psi ({\mathcal {L}})\!=\!\sigma _{{\text {ap}}}({\mathcal {L}})\!=\!{\mathbb {C}}. \end{aligned}$$

By the definition of $W_{\Psi ,2}^2({\mathcal {L}})$ and since $W_{\varepsilon ,2}^2({\mathcal {L}}) \!\subseteq \!B_\varepsilon (0)$, $\varepsilon \!>\!0$, it follows that $W_{\Psi ,2}^2({\mathcal {L}})=\{0\}$ which, together with (4.2), proves the first three equalities. To prove the two equalities on the right, and hence the claimed inequality, let $\uplambda \!\in \!{\mathbb {C}}$ be arbitrary. If $\uplambda \!=\!0$, then $\uplambda \in W^{2}_\Psi ({\mathcal {L}})$ by (4.3). If $\uplambda \!\ne \! 0$, we define the bounded operator matrices

$$\begin{aligned} {\mathcal {B}}_{k} {:}{=}\left( \begin{array}{c@{\quad }c} -{\text {diag}}(\frac{\uplambda }{m}\delta _{mk}:m\!\in \!{\mathbb {N}}) &{} 0 \\ -{\text {diag}}(\frac{\uplambda ^2}{m^2}\delta _{mk}:m\!\in \!{\mathbb {N}}) &{} {\text {diag}}(\frac{\uplambda }{m}\delta _{mk}:m\!\in \!{\mathbb {N}}) \end{array}\right) , \quad k \in {\mathbb {N}}, \end{aligned}$$

where $\delta _{mk}$ denotes the Kronecker delta. Then $\left\Vert {\mathcal {B}}_{k}\right\Vert \rightarrow 0$ as $k\rightarrow \infty$ and a straightforward calculation shows that

$$\begin{aligned} ({\mathcal {A}}-\uplambda I_{\mathcal {H}}) f_k \!=\!{\mathcal {B}}_{k} f_k , \quad f_k \!{:}{=}\!\frac{{\widetilde{f}}_k}{\Vert {\widetilde{f}}_k\Vert }\in {\text {dom}}{\mathcal {A}}, \quad {\widetilde{f}}_k \!=\! \left( {\begin{array}{c}\frac{k(k+1)}{\uplambda }e_{k} \\ e_{k}\end{array}}\right) , \quad k\in {\mathbb {N}}. \end{aligned}$$

On the one hand, for arbitrary $\varepsilon >0$, this implies that there exists $N\in {\mathbb {N}}$ such that $\left\Vert {\mathcal {B}}_N\right\Vert <\varepsilon$ and $0\in \sigma _{\mathrm{{p}}}({\mathcal {A}}-\uplambda I_{{\mathcal {H}}}-{\mathcal {B}}_N)=\sigma _{{\text {p}}}({\mathcal {L}}(\uplambda )-{\mathcal {B}}_N)$, whence

$$\begin{aligned} \uplambda \in \sigma _{{\text {p}}}({\mathcal {L}}-{\mathcal {B}}_N)\subseteq W^2({\mathcal {L}}-{\mathcal {B}}_N)\subseteq W_\varepsilon ^2({\mathcal {L}}) \end{aligned}$$

and thus $\uplambda \in W_\Psi ^2({\mathcal {L}})$ by intersection over all $\varepsilon >0$. On the other hand, $\uplambda \in \sigma _{{\text {ap}}}({\mathcal {L}})$ since the normalised sequence $\{f_k\}_{k}\subseteq {\text {dom}}{\mathcal {L}}(\uplambda )$ satisfies

$$\begin{aligned} \left\Vert ({\mathcal {A}}-\uplambda ) f_k \right\Vert =\left\Vert {\mathcal {B}}_{k}f_k\right\Vert \le \left\Vert {\mathcal {B}}_{k}\right\Vert \rightarrow 0, \quad k \rightarrow \infty . \end{aligned}$$

With one exception, we now focus on the most important case $n\!=\!2$ for which the notation

4.4 $$\begin{aligned} \begin{aligned}&{\mathcal {L}}(\uplambda ) \!{:}{=}\!\begin{pmatrix} A(\uplambda ) \!&{}\! B(\uplambda ) \\ C(\uplambda ) \!&{}\! D(\uplambda ) \end{pmatrix} \ \text{ in } {\mathcal {H}}={\mathcal {H}}_1\oplus {\mathcal {H}}_2, \\&{\text {dom}}{\mathcal {L}}(\uplambda ) \!{:}{=}\! \big ( {\text {dom}}A(\uplambda ) \cap {\text {dom}}C(\uplambda ) \big ) \oplus \big ( {\text {dom}}B(\uplambda ) \cap {\text {dom}}D(\uplambda ) \big ), \end{aligned} \end{aligned}$$

is more customary. We establish various inclusions between the (pseudo) quadratic numerical range $W^2_{(\Psi )}({\mathcal {L}})$ and the (pseudo) numerical ranges of the diagonal operator functions *A*, *D*, as well as between $W^2_{(\Psi )}({\mathcal {L}})$ and the (pseudo) numerical ranges of the Schur complements of ${\mathcal {L}}$.

### Proposition 4.6

(i) The quadratic numerical range and the pseudo quadratic numerical range satisfy
  $$\begin{aligned} W^2({\mathcal {L}})\subseteq W({\mathcal {L}}), \quad W^2_\Psi ({\mathcal {L}})\subseteq W_\Psi ({\mathcal {L}}). \end{aligned}$$
(ii) Let $\Omega _1:=\{\uplambda \in \Omega :{\mathcal {D}}_1(\uplambda )={\text {dom}}A(\uplambda )\}$ and suppose $\dim {\mathcal {H}}_2 >1$. Then
  $$\begin{aligned} W(A) \cap \Omega _1 \subseteq W^2({\mathcal {L}}), \quad W_\Psi (A) \cap \Omega _1 \subseteq W_{\Psi ,2}^2({\mathcal {L}}) \subseteq W_\Psi ^2({\mathcal {L}}); \end{aligned}$$
  if $\,{\mathcal {D}}_1(\uplambda )\!=\!{\text {dom}}A(\uplambda )$ for all $\uplambda \!\in \! W(A)$ or $\uplambda \!\in \! W_\Psi (A)$, respectively, then
  $$\begin{aligned} W(A)\subseteq W^2({\mathcal {L}}), \quad W_\Psi (A) \subseteq W_{\Psi ,2}^2({\mathcal {L}}) \subseteq W_\Psi ^2({\mathcal {L}}). \end{aligned}$$
(iii) Let $\Omega _2\!:=\!\{\uplambda \!\in \! \Omega :{\mathcal {D}}_2(\uplambda )\!=\!{\text {dom}}D(\uplambda )\}$ and suppose $\dim {\mathcal {H}}_1>1$. Then
  $$\begin{aligned} W(D) \cap \Omega _2 \subseteq W^2({\mathcal {L}}), \quad W_\Psi (D) \cap \Omega _2 \subseteq W_{\Psi ,2}^2({\mathcal {L}}) \subseteq W_\Psi ^2({\mathcal {L}}); \end{aligned}$$
  if $\,{\mathcal {D}}_2(\uplambda )\!=\!{\text {dom}}D(\uplambda )$ for all $\uplambda \!\in \! W(D)$ or $\uplambda \!\in \! W_\Psi (D)$, respectively, then
  $$\begin{aligned} W(D)\subseteq W^2({\mathcal {L}}), \quad W_\Psi (D) \subseteq W_{\Psi ,2}^2({\mathcal {L}}) \subseteq W_\Psi ^2({\mathcal {L}}). \end{aligned}$$

### Proof

The claims for the quadratic numerical range are consequences of (4.1) and of the corresponding statements [27,  Prop. 3.2, 3.3 (i),(ii)] for operator matrices. So it remains to prove the claims (i) and (ii) for the pseudo quadratic numerical range; the proof of claim (iii) is completely analogous.

(i) The inclusion for the quadratic numerical range in (i) applied to ${\mathcal {L}}\!+\!{\mathcal {B}}$ with $\left\Vert {\mathcal {B}}\right\Vert \!<\!\varepsilon$ yields $W^2_\varepsilon ({\mathcal {L}})\!\subseteq \! W_\varepsilon ({\mathcal {L}})$ for any $\varepsilon \!>\!0$. The claim for the pseudo quadratic numerical range follows if we take the intersection over all $\varepsilon \!>\!0$.

(ii) Let $\uplambda \!\in \! W_\varepsilon (A) \cap \Omega _1$ with $\varepsilon \!>\!0$ arbitrary. Then there exists a bounded operator $B_\varepsilon$ in ${\mathcal {H}}_1$ with $\left\Vert B_\varepsilon \right\Vert \!<\!\varepsilon$ and $\uplambda \!\in \! W(A+B_\varepsilon )$. Since ${\text {dom}}(A(\uplambda )+B_\varepsilon )$ $= {\text {dom}}A(\uplambda ) \subseteq {\text {dom}}C(\uplambda )$, the inclusion for the quadratic numerical range in (ii) applied to ${\mathcal {L}}+ {\text {diag}}(B_\varepsilon ,0_{{\mathcal {H}}_2})$ shows that

$$\begin{aligned} \uplambda \in W^2({\mathcal {L}}+{\text {diag}}(B_\varepsilon ,0_{{\mathcal {H}}_2})) \subseteq W_{\varepsilon ,2}^2({\mathcal {L}}) \subseteq W^2_\varepsilon ({\mathcal {L}}). \end{aligned}$$

By intersecting over all $\varepsilon >0$, we obtain $\uplambda \in W^2_{\Psi ,2} ({\mathcal {L}}) \subseteq W_\Psi ^2({\mathcal {L}})$. The second claim is obvious from the first one since then $\Omega _1 \subseteq W_\Psi (A)$. $\square$

Both qualitative and quantitative behaviour of operator matrices are closely linked to the properties of their so-called Schur complements, see e.g. [27]; the same is true for operator matrix functions, see e.g. [28] for the case of bounded operator values.

### Definition 4.7

The Schur complements of the $2\times 2$ operator matrix family ${\mathcal {L}}=\left\{ {\mathcal {L}}(\uplambda ):\uplambda \in \Omega \right\}$ in ${\mathcal {H}}={\mathcal {H}}_1\oplus {\mathcal {H}}_2$ as in (4.4) are the families

$$\begin{aligned} S_1(\uplambda )&{:}{=}A(\uplambda )-B(\uplambda )D(\uplambda )^{-1}C(\uplambda ), \quad \uplambda \in \rho (D), \\ S_2(\uplambda )&{:}{=}D(\uplambda )-C(\uplambda )A(\uplambda )^{-1}B(\uplambda ), \quad \uplambda \in \rho (A), \end{aligned}$$

of linear operators in ${\mathcal {H}}_1$ and ${\mathcal {H}}_2$, respectively, with domains

$$\begin{aligned} {\text {dom}}S_1(\uplambda )&{:}{=}\left\{ f\in {\mathcal {D}}_1(\uplambda ):D(\uplambda )^{-1}C(\uplambda )f\in {\text {dom}}B(\uplambda )\right\} , \quad \uplambda \in \rho (D), \\ {\text {dom}}S_2(\uplambda )&{:}{=}\left\{ f\in {\mathcal {D}}_2(\uplambda ):A(\uplambda )^{-1}B(\uplambda )f\in {\text {dom}}C(\uplambda )\right\} , \quad \uplambda \in \rho (A). \end{aligned}$$

The following inclusions between the numerical ranges and pseudo numerical ranges of the Schur complements $S_1$, $S_2$ and the quadratic numerical range and pseudo quadratic numerical range, respectively, of ${\mathcal {L}}$ hold.

### Proposition 4.8

The numerical ranges and pseudo numerical ranges of the Schur complements satisfy

$$\begin{aligned} W(S_1)\cup W(S_2)\subseteq W^2({\mathcal {L}}), \quad W_\Psi (S_1)\cup W_\Psi (S_2) \subseteq W_{\Psi ,2}^2({\mathcal {L}}) \subseteq W_\Psi ^2({\mathcal {L}}). \end{aligned}$$

### Proof

The first claim follows from (4.1) and the corresponding statement [26,  Thm. 2.5.8] for unbounded operator matrices.

Using the first claim, the second claim can be proven in a similar way as the claim for the pseudo numerical range in Proposition 4.6 (ii). $\square$

The following spectral enclosure properties of the block numerical range and pseudo block numerical range hold for operator matrix functions. They generalise results for the case of bounded operator values from [31], see also [28] for $n=2$, as well as the results for the operator function case, i.e. $n=1$, in Proposition 3.1.

### Proposition 4.9

Let ${\mathcal {L}}$ be a family of operator matrices. Then

$$\begin{aligned} \sigma _{{\text {p}}}({\mathcal {L}})\subseteq W^{n}({\mathcal {L}})\subseteq W_\Psi ^{n}({\mathcal {L}}). \end{aligned}$$

### Proof

The proof of the first inclusion is analogous to the bounded case, see [31,  Thm. 2.14] or [28,  Thm. 3.1] for $n\!=\!2$; the second inclusion is obvious, see Remark 4.2. $\square$

### Theorem 4.10

Let ${\mathcal {L}}$ be a family of operator matrices in ${\mathcal {H}}={\mathcal {H}}_1\oplus \dots \oplus {\mathcal {H}}_n$. For every $\varepsilon \!>\!0$,

4.5 $$\begin{aligned} \sigma _{\mathrm{ap},\varepsilon }({\mathcal {L}}) \subseteq W_\varepsilon ^n ({\mathcal {L}}), \qquad \left\Vert {\mathcal {L}}(\uplambda )^{-1}\right\Vert \le \frac{1}{\varepsilon }, \quad \uplambda \in \rho ({\mathcal {L}}){\setminus } W_\varepsilon ^n({\mathcal {L}}), \end{aligned}$$

and hence

$$\begin{aligned} \sigma _{{\text {ap}}}({\mathcal {L}})\subseteq W_\Psi ^n ({\mathcal {L}}); \end{aligned}$$

if, for all $\uplambda \in \Omega$, $\sigma ({\mathcal {L}}(\uplambda ))\subseteq \overline{W^n({\mathcal {L}}(\uplambda ))}$, then

$$\begin{aligned} \sigma ({\mathcal {L}})\subseteq W^n_{\Psi ,0}({\mathcal {L}}) \subseteq W_\Psi ^n ({\mathcal {L}}). \end{aligned}$$

### Proof

First let $\uplambda \!\in \! \sigma _{\mathrm{ap},\varepsilon }({\mathcal {L}})$. Then there exists $f_\varepsilon \!\in \!{\text {dom}}{\mathcal {L}}(\uplambda )$, $\left\Vert f_\varepsilon \right\Vert \!=\!1$, with $\left\Vert {\mathcal {L}}(\uplambda ) f_\varepsilon \right\Vert \!<\!\varepsilon$. The linear operator in ${\mathcal {H}}$ given by

$$\begin{aligned} {\mathcal {B}}f {:}{=}{\left\{ \begin{array}{ll} {\mathcal {L}}(\uplambda ) \mu f_\varepsilon &{} \mathrm{if}~f= \mu f_\varepsilon \in {\text {span}}f_\varepsilon , \\ \ \ \ \ 0 &{} \mathrm{if}~f \perp f_\varepsilon , \end{array}\right. } \end{aligned}$$

is bounded with $\left\Vert {\mathcal {B}}\right\Vert \!=\!\left\Vert {\mathcal {L}}(\uplambda )f_\varepsilon \right\Vert \!<\!\varepsilon$ and $({\mathcal {L}}(\uplambda )\!-\!{\mathcal {B}})f_\varepsilon \!=\!0$, i.e. $\uplambda \!\in \!\sigma _{{\text {p}}}({\mathcal {L}}\!-\!{\mathcal {B}})$. By Proposition 4.9 and since $\Vert {\mathcal {B}}\Vert \!<\!\varepsilon$, we conclude that $\uplambda \!\in \! W^n ({\mathcal {L}}-{\mathcal {B}})\!\subseteq \! W_\varepsilon ^n({\mathcal {L}})$, which proves the first claim.

The resolvent estimate in (4.5) follows from the first claim and from the definition of $\sigma _{\mathrm{{ap}},\varepsilon }({\mathcal {L}})$, cf. the proof of Proposition 3.1.

Taking the intersection over all $\varepsilon >0$ in the first claim, we obtain the inclusion $\sigma _{{\text {ap}}}({\mathcal {L}})\subseteq W_\Psi ^n({\mathcal {L}})$.

Finally, the assumption that $\sigma ({\mathcal {L}}(\uplambda ))\!\subseteq \!\overline{W^n({\mathcal {L}}(\uplambda ))}$ for all $\uplambda \!\in \!\Omega$ implies that $\sigma ({\mathcal {L}})\subseteq W^n_{\Psi ,0}({\mathcal {L}})$, see Definition 4.3. Now the second inequality in the last claim follows from the inclusion $W_{\Psi ,0}^n({\mathcal {L}})\!\subseteq \! W_\Psi ^n ({\mathcal {L}})$ by Proposition 4.4. $\square$

## Spectral Enclosures by Pseudo Numerical Ranges of Schur Complements

In this section we establish a new enclosure of the approximate point spectrum of an operator matrix family ${\mathcal {L}}$ by means of the pseudo numerical ranges of the associated Schur complements and hence, by Proposition 4.8, in $W^2_{\Psi ,2} ({\mathcal {L}})$ and in the pseudo quadratic numerical range $W_\Psi ^2({\mathcal {L}})$. Compared to earlier work, we no longer need restrictive dominance assumptions.

### Theorem 5.1

Suppose that ${\mathcal L}$ is a family of $2 \times 2$ operator matrices as in (4.4). If $\uplambda \in \sigma _{{\text {ap}}}({\mathcal {L}}){\setminus }(\sigma (A)\cup \sigma (D))$ is such that one of the conditions

(i) $C(\uplambda )$ is $A(\uplambda )$-bounded and $B(\uplambda )$ is $D(\uplambda )$-bounded;
(ii) $A(\uplambda )$ is $C(\uplambda )$-bounded, $D(\uplambda )$ is $B(\uplambda )$-bounded and both $C(\uplambda )$ and $B(\uplambda )$ are boundedly invertible;

is satisfied, then $\uplambda \in \sigma _{{\text {ap}}}(S_1)\cup \sigma _{{\text {ap}}}(S_2)$. If for all $\uplambda \in \rho (A)\cap \rho (D)$ one of the conditions (i) or (ii) is satisfied, then

5.1 $$\begin{aligned} \begin{aligned} \sigma _{{\text {ap}}}({\mathcal {L}}){\setminus }(\sigma (A)\cup \sigma (D))&\subseteq \sigma _{{\text {ap}}}(S_1)\cup \sigma _{{\text {ap}}}(S_2) \\&\subseteq W_\Psi (S_1)\cup W_\Psi (S_2) \subseteq W^2_{\Psi ,2} ({\mathcal {L}}) \subseteq W^2_\Psi ({\mathcal {L}}). \end{aligned} \end{aligned}$$

### Proof

Let $\uplambda \in \sigma _{{\text {ap}}}({\mathcal {L}})$. Then there exists a sequence $\{(u_n,v_n)\}_n\subseteq {\text {dom}}{\mathcal {L}}(\uplambda )$ with $\left\Vert u_n\right\Vert ^2+\left\Vert v_n\right\Vert ^2=1$, $n\in {\mathbb {N}}$, and

5.2 $$\begin{aligned} A(\uplambda )u_n+B(\uplambda )v_n {=}{:}h_n ~ \rightarrow ~0, \quad n \rightarrow \infty , \end{aligned}$$

5.3 $$\begin{aligned} C(\uplambda )u_n+D(\uplambda )v_n {=}{:}k_n ~ \rightarrow ~0, \quad n \rightarrow \infty . \end{aligned}$$

The normalisation implies that $\liminf _{n\rightarrow \infty }\left\Vert u_n\right\Vert \!>\!0$ or $\liminf _{n\rightarrow \infty }\left\Vert v_n\right\Vert \!>\!0$. Let $\liminf _{n\rightarrow \infty }\left\Vert u_n\right\Vert \!>\!0$, without loss of generality $\inf _{n\in {\mathbb {N}}}\left\Vert u_n\right\Vert \!>\!0$. We show that, if $\uplambda \in \rho (D)$, then $\uplambda \!\in \!\sigma _{{\text {ap}}}(S_1)$; if $\liminf _{n\rightarrow \infty }\left\Vert v_n\right\Vert \!>\!0$, an analogous proof yields that, if $\uplambda \in \rho (A)$, then $\uplambda \!\in \!\sigma _{{\text {ap}}}(S_2)$.

First we assume that $\uplambda$ satisfies (i). Since $\uplambda \in \rho (D)$, (5.3) implies that

$$\begin{aligned} v_n=D(\uplambda )^{-1}k_n-D(\uplambda )^{-1}C(\uplambda )u_n, \quad n\in {\mathbb {N}}. \end{aligned}$$

Inserting this into (5.2) and using ${\text {dom}}D(\uplambda )\subseteq {\text {dom}}B(\uplambda )$, we conclude that

5.4 $$\begin{aligned} S_1(\uplambda )u_n+B(\uplambda )D(\uplambda )^{-1}k_n=h_n ~ \rightarrow ~ 0, \quad n\rightarrow \infty . \end{aligned}$$

Due to (i) $B(\uplambda )D(\uplambda )^{-1}$ is bounded and hence $B(\uplambda )D(\uplambda )^{-1}k_n\rightarrow 0$, $n\rightarrow \infty$. Then (5.4) yields that $S_1(\uplambda )u_n\rightarrow 0$, $n\rightarrow \infty$. Because $\inf _{n\in {\mathbb {N}}}\left\Vert u_n\right\Vert >0$, we can set

$$\begin{aligned} f_n{:}{=}\frac{u_n}{\left\Vert u_n\right\Vert }\in {\mathcal {D}}_1(\uplambda )={\text {dom}}S_1(\uplambda ), \quad n\in {\mathbb {N}}, \end{aligned}$$

and obtain that $S_1(\uplambda )f_n\rightarrow 0$ for $n\rightarrow \infty$, which proves $\uplambda \in \sigma _{{\text {ap}}}(S_1)$.

Now assume that $\uplambda$ satisfies (ii). Since $C(\uplambda )$ is invertible, (5.3) shows that

5.5 $$\begin{aligned} u_n=C(\uplambda )^{-1}k_n-C(\uplambda )^{-1}D(\uplambda )v_n {=}{:}C(\uplambda )^{-1}k_n-w_n, \quad n\in {\mathbb {N}}, \end{aligned}$$

where $w_n{:}{=}C(\uplambda )^{-1}D(\uplambda )v_n\in {\text {dom}}S_1(\uplambda )$ for $n\in {\mathbb {N}}$ since

$$\begin{aligned} w_n\in {\mathcal {D}}_1(\uplambda )={\text {dom}}C(\uplambda ), \quad D(\uplambda )^{-1}C(\uplambda )w_n=v_n\in {\mathcal {D}}_2(\uplambda )={\text {dom}}B(\uplambda ). \end{aligned}$$

Inserting (5.5) into (5.2) and using ${\text {dom}}C(\uplambda )\subseteq {\text {dom}}A(\uplambda )$, we obtain that

5.6 $$\begin{aligned} A(\uplambda )C(\uplambda )^{-1}k_n-S_1(\uplambda )w_n=h_n ~ \rightarrow ~0, \quad n\rightarrow \infty . \end{aligned}$$

Since $C(\uplambda )^{-1}$ is bounded, we have $C(\uplambda )^{-1}k_n\!\rightarrow \!0$, $n\!\rightarrow \!\infty$. Thus $\inf _{n\in {\mathbb {N}}}\left\Vert u_n\right\Vert >0$ and (5.5) show that, without loss of generality, we can assume that $\inf _{n\in {\mathbb {N}}}\left\Vert w_n\right\Vert >0$. Set

$$\begin{aligned} g_n{:}{=}\frac{w_n}{\left\Vert w_n\right\Vert }\in {\text {dom}}S_1(\uplambda ), \quad n\in {\mathbb {N}}. \end{aligned}$$

By (ii) $A(\uplambda )C(\uplambda )^{-1}$ is bounded and so $A(\uplambda )C(\uplambda )^{-1}k_n\!\rightarrow \! 0$, $n\!\rightarrow \!\infty$. Now (5.6) yields $S_1(\uplambda )w_n\!\rightarrow \!0$ and thus $S_1(\uplambda )g_n \!\rightarrow \!0$, $n\!\rightarrow \!\infty$, which proves $\uplambda \!\in \!\sigma _{{\text {ap}}}(S_1)$.

Finally, the first inclusion in (5.1) is obvious from what was already shown; the second inclusion in (5.1) follows from Proposition 3.1 and the last two inclusions from Proposition 4.8. $\square$

### Remark 5.2

If, under the assumptions of Theorem 5.1, the Schur complements $S_1$ and $S_2$ satisfy the assumptions of Theorem 3.3 or 3.5 on every connected component of $\rho (D)$ and $\rho (A)$, respectively, then

$$\begin{aligned} \sigma _{{\text {ap}}}({\mathcal {L}}){\setminus }(\sigma (A)\cup \sigma (D))\subseteq \overline{W(S_1)}\cup \overline{W(S_2)}\subseteq \overline{W^2({\mathcal {L}})}, \end{aligned}$$

see Proposition 4.8 for the second inclusion.

For operator matrix families ${\mathcal {L}}$ with off-diagonal entries that are symmetric or anti-symmetric to each other, we now establish conditions ensuring that the approximate point spectrum of ${\mathcal {L}}$ is contained in the union of the approximate point spectrum of one Schur complement and the pseudo numerical range of the corresponding diagonal entry, i.e. $S_1$ and *D* or $S_2$ and *A*.

### Theorem 5.3

Let ${\mathcal {L}}$ be an operator matrix family as in (4.4).

(i) If $\,\uplambda \!\in \!\sigma _{{\text {ap}}}({\mathcal {L}})\!{\setminus }\!\sigma (D)$ is such that $C(\uplambda )\!\subseteq \! \pm B(\uplambda )^*\!$, $A(\uplambda )$ is accretive, ${\mp } D(\uplambda )$ sectorial with vertex 0 and $B(\uplambda )$ is $D(\uplambda )$-bounded, then $\uplambda \!\in \!\sigma _{{\text {ap}}}(S_1)\cup W_\Psi (D)$. If these conditions hold for all $\uplambda \!\in \!\rho (D)$, then
  5.7 $$\begin{aligned} \sigma _{{\text {ap}}}({\mathcal {L}})\!{\setminus }\!\sigma (D) \!\subseteq \! \sigma _{{\text {ap}}}(S_1)\!\cup \! W_\Psi (D) \!\subseteq \! W_\Psi (S_1)\cup W_\Psi (D); \end{aligned}$$
  if $$\dim {\mathcal {H}}_1 > 1$$, then
  5.8 $$\begin{aligned} \sigma _{{\text {ap}}}({\mathcal {L}})\!{\setminus }\!\sigma (D) \subseteq W_{\Psi ,2}^2({\mathcal {L}})\!\subseteq \! W_\Psi ^2({\mathcal {L}}). \end{aligned}$$
(ii) If $\uplambda \!\in \!\sigma _{{\text {ap}}}({\mathcal {L}})\!{\setminus }\!\sigma (A)$ is such that $C(\uplambda )\!\subseteq \! \pm B(\uplambda )^*\!$, $A(\uplambda )$ is sectorial with vertex 0, ${\mp } D(\uplambda )$ accretive and $C(\uplambda )$ is $A(\uplambda )$-bounded, then $\uplambda \!\in \!\sigma _{{\text {ap}}}(S_2)\cup W_\Psi (A)$. If these conditions hold for all $\uplambda \!\in \!\rho (A)$, then
  $$\begin{aligned} \sigma _{{\text {ap}}}({\mathcal {L}})\!{\setminus }\!\sigma (A) \!\subseteq \! \sigma _{{\text {ap}}}(S_2)\!\cup \! W_\Psi (A) \!\subseteq \! W_\Psi (S_2)\cup W_\Psi (A); \end{aligned}$$
  if $$\dim {\mathcal {H}}_2 > 1$$, then
  $$\begin{aligned} \sigma _{{\text {ap}}}({\mathcal {L}})\!{\setminus }\!\sigma (A) \subseteq W_{\Psi ,2}^2({\mathcal {L}})\!\subseteq \! W_\Psi ^2({\mathcal {L}}). \end{aligned}$$

Note that here we do not assume that the entries of ${\mathcal {L}}$ are holomorphic. In the next section Theorem 5.3 will be applied with $B(\uplambda ) = {\text {e}}^{\mathrm{{i}}\omega (\uplambda )} B$ and $C(\uplambda ) = {\text {e}}^{-\mathrm{{i}}\omega (\uplambda )} C$, where $C \subseteq B^*$ are constant and $\omega$ is real-valued, see the proof of Theorem 6.1.

The following corollary is immediate from Theorem 5.3 due to Proposition 4.6 and Proposition 4.8.

### Corollary 5.4

Under the assumptions of Theorem 5.3, if in (i) additionally $\sigma (D) \! \subseteq \! W_\Psi (D)$, then

$$\begin{aligned} \sigma _{{\text {ap}}}({\mathcal {L}}) \!\subseteq \! \sigma _{{\text {ap}}}(S_1)\!\cup \! W_\Psi (D) \!\subseteq \! W_\Psi (S_1)\cup W_\Psi (D) \!\subseteq \! W_{\Psi ,2}^2({\mathcal {L}})\!\subseteq \! W_\Psi ^2({\mathcal {L}}), \end{aligned}$$

and if in (ii) additionally $\sigma (A) \! \subseteq \! W_\Psi (A)$, then

$$\begin{aligned} \sigma _{{\text {ap}}}({\mathcal {L}}) \!\subseteq \! \sigma _{{\text {ap}}}(S_2)\!\cup \! W_\Psi (A) \!\subseteq \! W_\Psi (S_2)\cup W_\Psi (A) \!\subseteq \! W_{\Psi ,2}^2({\mathcal {L}})\!\subseteq \! W_\Psi ^2({\mathcal {L}}). \end{aligned}$$

### Proof of Theorem 5.3

We only prove (i); the proof of (ii) is analogous. Let $\uplambda \!\in \!\sigma _{{\text {ap}}}({\mathcal {L}})\!{\setminus }\!\sigma (D)$. In the same way as at the beginning of the proof of Theorem 5.1 we conclude that if $\liminf _{n\rightarrow \infty }\left\Vert u_n\right\Vert \!>\!0$, then $\uplambda \!\in \! \sigma _{{\text {ap}}}(S_1)$. It remains to be shown that in the case $\liminf _{n\rightarrow \infty }\left\Vert v_n\right\Vert \!>\!0$, without loss of generality $\inf _{n\in {\mathbb {N}}}\left\Vert v_n\right\Vert \!>\!0$, it follows that $\uplambda \!\in \! W_\Psi (D)$.

Taking the scalar product with $u_n$ in (5.2) and with $v_n$ in (5.3), respectively, we conclude that

5.9 $$\begin{aligned} (A(\uplambda )u_n,u_n) +(B(\uplambda )v_n,u_n)&=(h_n,u_n), \quad n\in {\mathbb {N}}, \end{aligned}$$

5.10 $$\begin{aligned} \pm (u_n,B(\uplambda )v_n) +(D(\uplambda )v_n,v_n)&=(k_n,v_n), \quad \, n\in {\mathbb {N}}. \end{aligned}$$

By subtracting from (5.9), or adding to (5.9), the complex conjugate of (5.10), we deduce that

$$\begin{aligned} (A(\uplambda )u_n,u_n) {\mp } \overline{(D(\uplambda )v_n,v_n)}=(h_n,u_n) {\mp } \overline{(k_n,v_n)}\rightarrow 0, \quad n\rightarrow \infty . \end{aligned}$$

Taking real parts and using the accretivity of $A(\uplambda )$ and ${\mp } D(\uplambda )$, we obtain

$$\begin{aligned} 0\le {\text {Re}}({\mp } D(\uplambda )v_n,v_n)\le {\text {Re}}(A(\uplambda )u_n,u_n){\mp }{\text {Re}}(D(\uplambda )v_n,v_n)\rightarrow 0, \quad n\rightarrow \infty . \end{aligned}$$

Since ${\mp }\! D(\uplambda )$ is sectorial with vertex 0 by assumption, this implies $({\mp } D(\uplambda )v_n,v_n)\! \rightarrow 0$ and hence $(D(\uplambda )v_n,v_n)\rightarrow 0$, $n\rightarrow \infty$, which proves that $\uplambda \!\in \! W_\Psi (D)$ by Proposition 2.3.

Finally, the first inclusion in (5.7) is obvious from what was already proved; the second inclusion in (5.7) follows from Proposition 3.1. The last claim in (5.8) is then a consequence of Propositions 4.6 (iii) and 4.8. $\square$

### Remark 5.5

(i) Sufficient conditions for the inclusions $\sigma (A)\! \subseteq \! W_\Psi (A)$ or $\sigma (D)\! \subseteq \! W_\Psi (D)$, respectively, may be found e.g. in Theorem 3.3 or Proposition 3.1.
(ii) An analogue of Remark 5.2 also holds for Theorem 5.3; the details of all possible combinations of assumptions and corresponding inclusions are left to the reader.

## Application to Structured Operator Matrices

In this section, we apply the results of the previous section to prove new spectral enclosures and resolvent estimates for non-selfadjoint operator matrix functions exhibiting a certain dichotomy.

More precisely, we consider a linear monic family ${\mathcal {L}}(\uplambda )={\mathcal {A}}-\uplambda I_{\mathcal {H}}$, $\uplambda \in {\mathbb {C}}$, with a densely defined operator matrix

6.1 $$\begin{aligned} {\mathcal {A}}\!=\!\left( \begin{array}{cc} A &{} B \\ C &{} D \end{array}\right) , \quad {\text {dom}}{\mathcal {A}}\!=\! \big ( {\text {dom}}A \cap {\text {dom}}C \big ) \!\oplus \! \big ( {\text {dom}}B \cap {\text {dom}}D \big ) \end{aligned}$$

with $C\!\subseteq \! B^*$ in ${\mathcal {H}}\!=\!{\mathcal {H}}_1\oplus {\mathcal {H}}_2$. We assume that the entries of ${\mathcal {A}}$ are densely defined closable linear operators acting between the respective spaces ${\mathcal {H}}_1$ and/or ${\mathcal {H}}_2$, and that *A*, $-D$ are accretive or even sectorial with vertex 0. This means that their numerical ranges lie in closed sectors $\Sigma _\omega$ with semi-axis ${\mathbb {R}}_+$ and semi-angle $\omega = \pi /2$ or $\omega \in [0,\pi /2)$, respectively, given by

$$\begin{aligned} \Sigma _\omega {:}{=}\left\{ z\in {\mathbb {C}}:\left|\arg z\right|\le \omega \right\} , \quad \omega \in [0,\pi /2]; \end{aligned}$$

here $\arg :{\mathbb {C}}\rightarrow (-\pi ,\pi ]$ is the argument of a complex number with $\arg 0=0$.

The next theorem no longer requires bounds on the dominance orders among the entries in the columns of ${\mathcal {A}}$, in contrast to earlier results in [27,  Thm. 5.2] where the relative bounds had to be 0.

### Theorem 6.1

Let ${\mathcal {A}}$ be an operator matrix as in (6.1) with $C\subseteq B^*$. Assume that there exist $\alpha$, $\delta \in {\mathbb {R}}$ and semi-angles $\varphi ,\psi \in [0,\pi /2]$ with

6.2 $$\begin{aligned} {\text {Re}}W(D)\le \delta<0<\alpha \le {\text {Re}}W(A), \quad W(A)\subseteq \Sigma _\varphi , \quad W(D)\subseteq -\Sigma _\psi . \end{aligned}$$

Suppose further that one of the following holds:

(i) *A*, $-D$ are m-accretive, *C* is *A*-bounded, *B* is *D*-bounded,
(ii) *A*, $-D$ are m-accretive, *A* is *C*-bounded, *D* is *B*-bounded and *B*, *C* are boundedly invertible,
(iii) $-D$ is m-sectorial with vertex 0, i.e. $\psi \!<\!\pi /2$, and *B* is *D*-bounded,
(iv) *A* is m-sectorial with vertex 0, i.e. $\varphi \!<\!\pi /2$, and *C* is *A*-bounded.

**Fig. 1:**
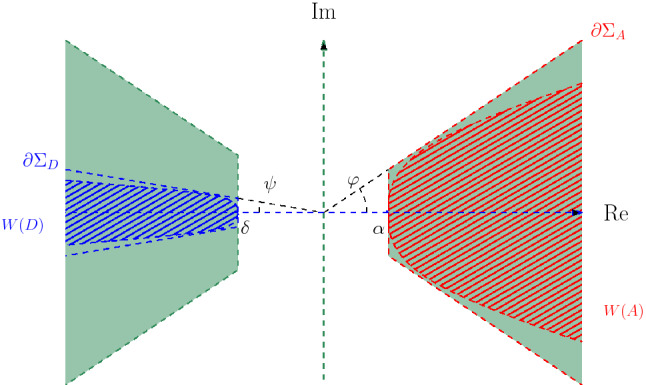
The set $\Sigma$ (green) enclosing $\sigma _{{\text {ap}}}({\mathcal {A}})$, see (6.3) ; inside the sets $\Sigma _A\!{:}{=}\! \Sigma _\varphi \!{\setminus }\! S$ (bounded by red line) enclosing *W*(*A*) (red, dashed) and $\Sigma _D\!{:}{=}\! -\Sigma _\psi \!{\setminus }\! S$ (bounded by blue line ) enclosing *W*(*D*) (blue, dashed), separated by $S\!{:}{=}\! \{z\!\in \!{\mathbb {C}}:{\text {Re}}z\!\in \! (\delta ,\alpha )\}$, see (6.2) (color figure online)

Then, with $$\tau {:}{=}\max \{\varphi ,\psi \}$$,

6.3 $$\begin{aligned} \sigma _{{\text {ap}}}({\mathcal {A}})\subseteq (-\Sigma _\tau \cup \Sigma _\tau )\cap \left\{ z\in {\mathbb {C}}:{\text {Re}}z\notin (\delta ,\alpha )\right\} {=}{:}\Sigma ; \end{aligned}$$

if, in addition, $\rho ({\mathcal {A}})\cap \Sigma ^{{\text {c}}}\ne \emptyset$, then $\sigma ({\mathcal {A}})\subseteq \Sigma$. Figure 1 illustrates the enclosure (6.3) of $\sigma _p({\mathcal A})$ in terms of the numerical ranges of the diagonal elements *A* and *D* in (6.2).

The proof of Theorem 6.1 relies on Theorems 5.1 and 5.3, and on the following enclosures for the pseudo numerical ranges of the Schur complements.

### Lemma 6.2

Let ${\mathcal {A}}$ be as in (6.1) with $C\!\subseteq \!B^*$ and let $\uplambda \in {\mathbb {C}}$.

(i) Suppose *A*, $-D$ are uniformly accretive,
  6.4 $$\begin{aligned} {\text {Re}}W(D)\le \delta<0<\alpha \le {\text {Re}}W(A). \end{aligned}$$
  If $$\,{\text {Re}}\uplambda \in (\delta ,\alpha )$$, then
  $$\begin{aligned} \begin{aligned} \uplambda \in \rho (D)&\implies {\text {Re}}\overline{W(S_1(\uplambda ))}\ge \alpha -{\text {Re}}\uplambda >0, \\ \uplambda \in \rho (A)&\implies {\text {Re}}\overline{W(S_2(\uplambda ))}\le \delta -{\text {Re}}\uplambda <0. \end{aligned} \end{aligned}$$
(ii) Suppose *A*, $-D$ are sectorial with vertex 0,
  $$\begin{aligned} W(A)\subseteq \Sigma _\varphi , \qquad W(D)\subseteq -\Sigma _\psi \end{aligned}$$
  with $\varphi ,\psi \!\in \![0,\pi /2)$ and let $\tau \!{:}{=}\!\max \{\varphi ,\psi \}$. If $\,\arg \uplambda \!\in \!(\tau ,\pi -\tau )$, then
  $$\begin{aligned} \begin{aligned} \uplambda \in \rho (D)&\ \implies \ \arg (\overline{W(S_1(\uplambda ))}+\uplambda ) \in [-\arg \uplambda ,\tau ], \\ \uplambda \in \rho (A)&\ \implies \ \arg (\overline{W(S_2(\uplambda ))}+\uplambda ) \in (\!-\!\pi ,-\arg \uplambda ]\cup [\pi -\tau ,\pi ]; \end{aligned} \end{aligned}$$
  if $$\,\arg \uplambda \!\in \!(-\pi +\tau ,-\tau )$$, then
  $$\begin{aligned} \begin{aligned} \uplambda \in \rho (D)&\ \implies \ \arg (\overline{W(S_1(\uplambda ))}+\uplambda ) \in [-\tau ,-\arg \uplambda ], \\ \uplambda \in \rho (A)&\ \implies \ \arg (\overline{W(S_2(\uplambda ))}+\uplambda ) \in (\!-\!\pi ,-\pi +\tau ]\cup [-\arg \uplambda ,\pi ]. \end{aligned} \end{aligned}$$

### Proof

We show the claims for $S_1$, the proofs for $S_2$ are analogous. It is easy to see that it suffices to prove the claimed non-strict inequalities for $W(S_1(\uplambda ))$. Let $\uplambda \in \rho (D)$, $f\in {\text {dom}}S_1(\uplambda )\subseteq {\text {dom}}A\cap {\text {dom}}B^*$ with $\left\Vert f\right\Vert =1$, and set $g{:}{=}(D-\uplambda )^{-1}B^*f$. Then

6.5 $$\begin{aligned} (S_1(\uplambda )f,f)=(Af,f)-\uplambda -\overline{(Dg,g)}+{\overline{\uplambda }} \left\Vert g\right\Vert ^2. \end{aligned}$$

(i) If ${\text {Re}}\uplambda \,\in (\delta ,\alpha )$, then (6.5) and (6.4) show that

$$\begin{aligned} {\text {Re}}(S_1(\uplambda )f,f)\ge \alpha -{\text {Re}}\uplambda +(-\delta +{\text {Re}}\uplambda )\left\Vert g\right\Vert ^2\ge \alpha -{\text {Re}}\uplambda >0. \end{aligned}$$

(ii) We consider $\arg \uplambda \!\in \!(\tau ,\pi \!-\!\tau )$, the case $\arg \uplambda \!\in \!(-\pi \!+\!\tau ,-\tau )$ can be shown analogously. By assumption, $|\arg (Af,f)|\!\le \!\varphi \!\le \!\tau$, $|\arg \overline{(-Dg,g)}|\!\le \!\psi \!\le \!\tau$. Together with $\arg ({\overline{\uplambda }}\left\Vert g\right\Vert ^2)=-\arg \uplambda \!\in \!(-\pi \!+\!\tau ,-\tau )$, it follows from (6.5) that

$$\begin{aligned} \arg \big ( (S_1(\uplambda )f,f)\!+\!\uplambda \big ) \!=\! \arg \big ((Af,f)\!+\!\overline{(-Dg,g)}\!+\!{\overline{\uplambda }}\left\Vert g\right\Vert ^2\big )\in [-\arg \uplambda ,\tau ]. \end{aligned}$$

$$\square $$

### Proof of Theorem 6.1

First we use Lemma 6.2 to show that if *A* or $-D$ are m-accretive, respectively, then

6.6 $$\begin{aligned} W_\Psi (S_2)\subseteq \Sigma \quad \mathrm {or} \quad W_\Psi (S_1)\subseteq \Sigma . \end{aligned}$$

We prove the claim for $S_1$ by taking complements; the proof for $S_2$ is analogous. To this end, let $\uplambda \in \Sigma ^{{\text {c}}} \subseteq \rho (D)$. Then ${\text {Re}}\uplambda \in (\delta ,\alpha )$ or $\left|\arg \uplambda \right|\in (\tau ,\pi -\tau )$; note that the latter case only occurs if both *A* and $-D$ are sectorial with vertex 0, i.e. if $\tau < \pi /2$. If ${\text {Re}}\uplambda \in (\delta ,\alpha )$, Lemma 6.2 (i) implies $0\notin \overline{W(S_1(\uplambda ))}$, i.e. $\uplambda \notin W_\Psi (S_1)$ by (2.2). In the same way, if $\left|\arg \uplambda \right|\in (\tau ,\pi -\tau )$, then $\uplambda \notin W_\Psi (S_1)$ follows from Lemma 6.2 (ii); indeed, otherwise we would have $0\in \overline{W(S_1(\uplambda ))}$ and hence, e.g. if $\arg \uplambda \in (\tau , \pi - \tau )$,

$$\begin{aligned} \arg (0 + \uplambda ) = \arg \uplambda \in [-\arg \uplambda , \tau ] \cap (\tau , \pi - \tau ) = \emptyset , \end{aligned}$$

and analogously for $\arg \uplambda \in (-\pi + \tau ,- \tau )$. This completes the proof of (6.6).

We show that assumptions (i) or (iii) imply (6.3); the proof when assumptions (ii) or (iv) hold is analogous.

Assume first that (i) holds and let $\uplambda \in \sigma _{{\text {ap}}}({\mathcal {A}})$. If $\uplambda \in \sigma (A)\cup \sigma (D)\subseteq \Sigma$, there is nothing to show. If $\uplambda \notin \sigma (A)\cup \sigma (D)$, then Theorem 5.1 (i) shows that $\uplambda \in W_\Psi (S_1)\cup W_\Psi (S_2)$ and we conclude $\uplambda \in \Sigma$ from (6.6).

Now assume that (iii) is satisfied. Then $-D$ is m-sectorial with vertex 0 and $\sigma (D) \subseteq \overline{W(D)}\subseteq \Sigma$. In order to prove (6.3), we show $\sigma _{{\text {ap}}}({\mathcal {A}}) \cap \Sigma ^{{\text {c}}} = \emptyset$. To this end, it suffices to prove that

6.7 $$\begin{aligned} \sigma _{{\text {ap}}}({\mathcal {A}}) \cap \Sigma ^{{\text {c}}}\subseteq W_\Psi (S_1) \cup W_\Psi (D-\cdot I_{{\mathcal {H}}_2}); \end{aligned}$$

here, in the sequel, we write $D-\cdot I_{{\mathcal {H}}_2}$ for the operator family $D - \uplambda I_{{\mathcal {H}}_2}$, $\uplambda \in {\mathbb {C}}$. Indeed, if (6.7) holds, then $W_\Psi (D-\cdot I_{{\mathcal {H}}_2}) = \overline{W(D)} \subseteq \Sigma$ and (6.6) yield that $\sigma _{{\text {ap}}}({\mathcal {A}}) \cap \Sigma ^{{\text {c}}} \subseteq \Sigma$ and hence the claim.

For the proof of (6.7), we will use Theorem 5.3 (i). To this end, for $\uplambda \in \Sigma ^{{\text {c}}}$, we define a rotation angle

$$\begin{aligned} \omega (\uplambda ) {:}{=}{\left\{ \begin{array}{ll} 0, &{} {\text {Re}}\uplambda \in (\delta , \alpha ), \\ {\text {sgn}}(\arg \uplambda ) \big |\frac{\pi }{2} - |\arg \uplambda |\big |, &{} {\text {Re}}\uplambda \notin (\delta , \alpha ) \wedge |\arg \uplambda | \in (\tau , \pi -\tau ); \end{array}\right. } \end{aligned}$$

note that the second case only occurs if *A* is sectorial with vertex 0, i.e. if $\tau < \pi /2$, and that then $\uplambda \ne 0$ and $|\omega (\uplambda )| \in (0,\pi /2-\tau )$. Define a rotated operator matrix family ${\widetilde{{\mathcal {L}}}}$ by

$$\begin{aligned} {\widetilde{{\mathcal {L}}}}(\uplambda ) \!{:}{=}\! {\text {diag}}\big (\!{\text {e}}^{\mathrm{{i}}\omega (\uplambda )}{\mathcal {I}}_{{\mathcal {H}}_1}, {\text {e}}^{-\mathrm{{i}}\omega (\uplambda )}{\mathcal {I}}_{{\mathcal {H}}_2}\!\big ) ({\mathcal {A}}-\uplambda {\mathcal {I}}_{\mathcal {H}}), \ \ {\text {dom}}{\widetilde{{\mathcal {L}}}} (\uplambda ) \!{:}{=}\! {\text {dom}}{\mathcal {A}}, \quad \uplambda \!\in \!\Sigma ^{{\text {c}}}\!. \end{aligned}$$

Since, for fixed $\uplambda \!\in \! \Sigma ^{{\text {c}}}$, the operator matrix ${\text {diag}}({\text {e}}^{\mathrm{{i}}\omega (\uplambda )}{\mathcal {I}}_{{\mathcal {H}}_1}, {\text {e}}^{-\mathrm{{i}}\omega (\uplambda )}{\mathcal {I}}_{{\mathcal {H}}_2})$ is bounded and boundedly invertible (even unitary), it is straightforward to show that

$$\begin{aligned} \uplambda \in \sigma _{{\text {ap}}}({\mathcal {A}}) \, \iff \, 0 \in \sigma _{{\text {ap}}}({\widetilde{{\mathcal {L}}}} (\uplambda )), \end{aligned}$$

which implies $\sigma _{{\text {ap}}}({\widetilde{{\mathcal {L}}}}) = \sigma _{{\text {ap}}}({\mathcal {A}}) \cap \Sigma ^{{\text {c}}}$. Moreover, the angle $\omega (\uplambda )$ is chosen such that ${\text {e}}^{\mathrm{{i}}\omega (\uplambda )}(A -\uplambda I_{{\mathcal {H}}_1})$ is accretive, $-{\text {e}}^{-\mathrm{{i}}\omega (\uplambda )}(D -\uplambda I_{{\mathcal {H}}_2})$ is sectorial with vertex 0 and ${\text {e}}^{-\mathrm{{i}}\omega (\uplambda )}C \!\subseteq \! {\text {e}}^{\mathrm{{i}}\omega (\uplambda )}B^*$ for every $\uplambda \!\in \! \Sigma ^{{\text {c}}}$. In fact, if ${\text {Re}}\uplambda \in (\delta , \alpha )$, this is obvious. If ${\text {Re}}\uplambda \notin (\delta , \alpha )$ and $|\arg \uplambda | \in (\tau , \pi -\tau )$, then $\varphi < \pi /2$ and $|\omega (\uplambda )| < \pi /2-\tau$ as mentioned above. From ${\text {Re}}W(A) \ge \alpha >0$ and $W(A) \subseteq \Sigma _\varphi$, it thus follows that ${\text {e}}^{\mathrm{{i}}\omega (\uplambda )}A$ is uniformly accretive and sectorial with vertex 0 and, since ${\text {Re}}({\text {e}}^{\mathrm{{i}}\omega (\uplambda )}\uplambda ) \le 0$ , the claim for ${\text {e}}^{\mathrm{{i}}\omega (\uplambda )}(A -\uplambda I_{{\mathcal {H}}_1})$ holds. The proof for $-{\text {e}}^{-\mathrm{{i}}\omega (\uplambda )}(D -\uplambda I_{{\mathcal {H}}_2})$ is analogous.

Thus ${\widetilde{{\mathcal {L}}}}$ satisfies the assumptions of Theorem 5.3 (i) and, because $\sigma ({\text {e}}^{-\mathrm{{i}}\omega }(D -\cdot I_{{\mathcal {H}}_2})) = \sigma (D) \cap \Sigma ^{{\text {c}}} = \emptyset$, (5.7) therein yields that

$$\begin{aligned} \sigma _{{\text {ap}}}({\mathcal {A}})\cap \Sigma ^{{\text {c}}} = \sigma _{{\text {ap}}}({\widetilde{{\mathcal {L}}}}) \subseteq W_\Psi ({\widetilde{S}}_1) \cup W_\Psi ({\text {e}}^{-\mathrm{{i}}\omega }(D -\cdot I_{{\mathcal {H}}_2})), \end{aligned}$$

where ${\widetilde{S}}_1$ is the first Schur complement of ${\widetilde{{\mathcal {L}}}}$. Now the claim (6.7) follows from the above inclusion and from the fact that, since ${\text {e}}^{\mathrm{{i}}\omega (\uplambda )}\!\ne \! 0$,

$$\begin{aligned} 0 \!\in \! \overline{W({\widetilde{S}}_1 (\uplambda ))} \!\iff \, 0 \!\in \! \overline{W({\text {e}}^{\mathrm{{i}}\omega (\uplambda )} S_1 (\uplambda ))} \!=\! {\text {e}}^{\mathrm{{i}}\omega (\uplambda )} \overline{W(S_1 (\uplambda ))} \iff 0 \!\in \! \overline{W(S_1 (\uplambda ))} \end{aligned}$$

for $\uplambda \!\in \! \Sigma ^{{\text {c}}}\!$, and analogously for the family ${\text {e}}^{-\mathrm{{i}}\omega }(D -\cdot I_{{\mathcal {H}}_2})$. This completes the proof that (i) and (iii) imply (6.3).

Finally, if $\rho ({\mathcal {A}})\cap \Sigma ^{{\text {c}}}\ne \emptyset$, then ${\mathcal {A}}$ is closed and $\sigma ({\mathcal {A}}) \!\subseteq \!\Sigma$ follows from $\sigma _{{\text {ap}}}({\mathcal {A}})\subseteq \Sigma$, see (6.3), and from the stability of Fredholm index, see [17,  Thm. IV.5.17]. $\square$

In Proposition 6.5 below, we derive sufficient conditions for $\rho ({\mathcal {A}})\cap \Sigma ^{{\text {c}}}\ne \emptyset$ in Theorem 6.1 for diagonally dominant and off-diagonally dominant operator matrices. For the latter, we use a result of [6], while for the former we employ the following lemma, inspired by an estimate in [17,  Prob. V.3.31] for accretive operators.

### Lemma 6.3

Let the linear operator *T* in ${\mathcal {H}}$ be m-sectorial with vertex 0 or m-accretive, i.e. there exists $\omega \!\in \!\left[ 0,\pi /2\right)$ or $\omega = \pi /2$, respectively, with $\sigma (T)\!\subseteq \!\overline{W(T)}\!\subseteq \!\Sigma _\omega$. Then

$$\begin{aligned} \left\Vert T(T\!-\!\uplambda )^{-1}\right\Vert \!\le \! \frac{1}{m_T(\arg \uplambda )}\!:=\! \left\{ \begin{array}{cl} \displaystyle \!\!\frac{1}{\sin (\left|\arg \uplambda \right|\!-\!\omega )},\! &{} \quad \left|\arg \uplambda \right|\!\in \!(\omega ,\omega \!+\!\frac{\pi }{2}), \\ \!\!1, &{} \quad \left|\arg \uplambda \right|\!\in \![\omega \!+\!\frac{\pi }{2},\pi ], \end{array}\right. \qquad \!\uplambda \!\notin \!\Sigma _\omega . \end{aligned}$$

### Proof

Let $\uplambda \notin \Sigma _\omega$ and $\varepsilon \in (0,\left|\uplambda \right|)$ be arbitrary. Then $\uplambda \in \rho (T)$, $-\varepsilon \in \rho (T)$, $\uplambda \ne -\varepsilon$ and we can write

6.8 $$\begin{aligned} T(T-\uplambda )^{-1}&=(T+\varepsilon )(T+\varepsilon -(\uplambda +\varepsilon ))^{-1}-\varepsilon (T-\uplambda )^{-1}, \nonumber \\&=-(\uplambda +\varepsilon )^{-1}\left( (T+\varepsilon )^{-1}-(\uplambda +\varepsilon )^{-1}\right) ^{-1}-\varepsilon (T-\uplambda )^{-1}. \end{aligned}$$

Since $\varepsilon >0$, it is easy to see that $T+\varepsilon$ is m-accretive or m-sectorial with semi-angle $\omega$ and vertex 0, and hence so is $(T+\varepsilon )^{-1}$, cf. [17,  Prob. .3.31] for the m-accretive case. Thus, by [17,  Thm. .3.2] and (6.8), we can estimate

$$\begin{aligned} \left\Vert T(T-\uplambda )^{-1}\right\Vert \le \frac{\left|\uplambda +\varepsilon \right|^{-1}}{{\text {dist}}\left( (\uplambda +\varepsilon )^{-1},\Sigma _\omega \right) }+\frac{\varepsilon }{{\text {dist}}\left( \uplambda ,\Sigma _\omega \right) }. \end{aligned}$$

The claim now follows by taking the limit $\varepsilon \rightarrow 0$ and using the estimate

6.9 $$\begin{aligned} {\text {dist}}\left( \uplambda ^{-1},\Sigma _\omega \right) \ge \left\{ \begin{array}{cl} \displaystyle \frac{\sin (\left|\arg \uplambda \right|-\omega )}{\left|\uplambda \right|}, &{} \,\left|\arg \uplambda \right|\in \left( \omega ,\omega +\frac{\pi }{2}\right) , \\ \displaystyle \frac{1}{\left|\uplambda \right|}, &{} \,\left|\arg \uplambda \right|\in \left[ \omega +\frac{\pi }{2},\pi \right] , \end{array}\right. \end{aligned}$$

cf. [16,  Thm. 2.2]. $$\square $$

### Remark 6.4

The inequality in Lemma 6.3 is optimal, equality is achieved e.g. for normal operators with spectrum on the boundary of $\Sigma _\omega$.

### Proposition 6.5

Suppose that, under the assumptions of Theorem 6.1, we strengthen assumptions (i) and (ii) to

(i$${'})$$
*A*, $-D$ are m-sectorial with vertex 0, i.e. $\varphi$, $\psi \!<\!\pi /2$ in (6.2), *C* is *A*-bounded with relative bound $\delta _A$ and *B* is *D*-bounded with relative bound $\delta _D$ such that
  $$\begin{aligned} \delta _A\delta _D&< \sin (\theta _{0}-\varphi )\sin (\theta _{0}+\psi ) =: M_{\theta _0} \in (0,1] \end{aligned}$$
  where
  $$\begin{aligned} \theta _0:= {\left\{ \begin{array}{ll} \max \big \{ \frac{\pi }{2} \!+\! \frac{\varphi -\psi }{2}, \tau \big \}, &{} \ \varphi \le \psi , \\ \,\min \big \{ \frac{\pi }{2} \!+\! \frac{\varphi -\psi }{2}, \pi \!-\!\tau \big \}, &{} \ \psi < \varphi ; \end{array}\right. } \end{aligned}$$
(ii$${'})$$
*A*, $-D$ are m-accretive, $C\!=\!B^*$, *A* is *C*-bounded with relative bound $\delta _C$, *D* is *B*-bounded with relative bound $\delta _B$ with
  $$\begin{aligned} \delta _B \delta _C < 1, \end{aligned}$$
  *B*, *C* are boundedly invertible, and the relative boundedness constants $a_C$, $a_B \!\ge \! 0$, $b_C$, $b_B \!\ge \! 0$ in
  $$\begin{aligned}&\Vert Ax\Vert ^2\le a_C^2\Vert x\Vert ^2+b_C^2\Vert Cx\Vert ^2, \quad x\in {\text {dom}}C, \\&\Vert Dy\Vert ^2\le a_B^2\Vert y\Vert ^2+b_B^2\Vert By\Vert ^2,\; \, \ y \in {\text {dom}}B, \end{aligned}$$
  satisfy
  $$\begin{aligned} \sqrt{a_C^2\Vert B^{-1}\Vert ^2+b_C^2} \sqrt{a_B^2\Vert B^{-1}\Vert ^2+b_B^2}<1. \end{aligned}$$

Then $\rho ({\mathcal {A}})\cap \Sigma ^{{\text {c}}}\ne \emptyset$ and hence

$$\begin{aligned} \sigma ({\mathcal {A}})\subseteq (-\Sigma _\tau \cup \Sigma _\tau )\cap \left\{ z\in {\mathbb {C}}:{\text {Re}}z\notin (\delta ,\alpha )\right\} = \Sigma . \end{aligned}$$

### Proof

By Theorem 6.1, it suffices to show $\rho ({\mathcal {A}})\cap \Sigma ^{{\text {c}}}\ne \emptyset$.

Suppose that (i${'}$) holds and let $\uplambda \!=\!r{\text {e}}^{\mathrm{{i}}\theta }$ with $r\!>\!0$, $\theta \!\in \! (\tau ,\pi \!-\!\tau )$ to be chosen later. Then $\uplambda \!\in \! \rho (A) \cap \rho (D)$. Since $\frac{1}{M_{\theta _0}} \delta _A \delta _D \!<\! 1$, there exists $\varepsilon \!>\!0$ so that

6.10 $$\begin{aligned} \frac{1}{M_{\theta _0} - \varepsilon } (\delta _A + \varepsilon ) (\delta _D + \varepsilon ) < 1. \end{aligned}$$

Due to the relative boundedness assumption on *C*, there exist $a_A$, $b_A>0$, $b_A\in [\delta _A,\delta _A+\varepsilon )$ such that

6.11 $$\begin{aligned} \left\Vert C(A-\uplambda )^{-1}\right\Vert \le a_A\left\Vert (A-\uplambda )^{-1}\right\Vert +b_A\left\Vert A(A-\uplambda )^{-1}\right\Vert . \end{aligned}$$

Since *A* is m-sectorial with semi-angle $\varphi$ and vertex 0, we have the estimate

6.12 $$\begin{aligned} \left\Vert (A-\uplambda )^{-1}\right\Vert \le \frac{1}{{\text {dist}}(\uplambda ,W(A))}\le \frac{1}{r m_A(\theta )}, \end{aligned}$$

with $m_{A} (\theta )$ defined as in Lemma 6.3, see [17,  Thm. V.3.2] or (6.9). Consequently, by (6.11), (6.12) and Lemma 6.3, we obtain

$$\begin{aligned} \left\Vert C(A-\uplambda )^{-1}\right\Vert \le \frac{a_A}{r m_A(\theta )} +\frac{b_A}{m_A(\theta )}. \end{aligned}$$

Similarly, since $-D$ is m-sectorial with semi-angle $\psi$ and vertex 0, and using Lemma 6.3 as well as (6.9) and $|\arg (-\uplambda )| = \pi -\theta$, we conclude that there exist $a_D$, $b_D>0$, $b_D\in [ \delta _D,\delta _D+\varepsilon )$ with

$$\begin{aligned} \left\Vert B(D-\uplambda )^{-1}\right\Vert \le \frac{a_D}{rm_{-D} (\pi -\theta )}+\frac{b_D}{{m_{-D}(\pi -\theta )}} \end{aligned}$$

with $m_{-D}(\pi -\theta )$ defined as in Lemma 6.3 and hence

6.13 $$\begin{aligned} \Vert C(A-\uplambda )^{-1} B(D-\uplambda )^{-1} \Vert \!\le \! \frac{b_A b_D}{M_\theta \!} \Big ( \frac{a_A}{r b_A} \!+\! 1 \Big ) \Big ( \frac{a_D}{r b_D} \!+\! 1 \Big ). \end{aligned}$$

Here the function

$$\begin{aligned}{}[\varphi , \pi -\psi ] \rightarrow [0,1], \quad \theta \mapsto M_\theta {:}{=}m_A(\theta ) m_{-D}(\pi -\theta ), \end{aligned}$$

is continuous, monotonically increasing for $\theta \le {\widetilde{\theta }}_0 := \frac{\pi }{2} + \frac{\varphi -\psi }{2} \in [\varphi , \pi -\psi ]$ and decreasing for $\theta \ge {\widetilde{\theta }}_0$. Hence, the restriction of $\theta \mapsto M_\theta$ to $[\tau , \pi -\tau ]$ attains its maximum at $\theta _0$ and we can choose $\delta >0$ such that $M_{\theta _0} - \varepsilon < M_\theta$ for $\theta \in (\theta _0-\delta ,\theta _0+\delta ) \cap (\tau ,\pi -\tau )$. Now we fix such a $\theta$. Using (6.13) and (6.10), we conclude that there exists $r>0$ so large that

$$\begin{aligned} \Vert C(A-\uplambda )^{-1} B(D-\uplambda )^{-1} \Vert \!\le \! \frac{(\delta _A+\varepsilon )(\delta _D+\varepsilon )}{M_{\theta _0}\!\!-\!\varepsilon } \Big ( \frac{a_A}{r b_A } \!+\! 1 \Big ) \Big ( \frac{a_D}{r b_D}\!+\! 1\Big ) \!<\! 1.\! \end{aligned}$$

This implies $1 \!\in \! \rho ( C(A\!-\!\uplambda )^{-1} B(D\!-\!\uplambda )^{-1})\!$ and thus $\uplambda \!\in \! \rho ({\mathcal {A}})$ by [26,  Cor. 2.3.5].

Suppose that (ii${'}$) is satisfied. By the assumptions on *B*, *C*, the operator ${\mathcal {S}}\!{:}{=}\!{\mathcal {S}}_1$ is selfadjoint and has a spectral gap $(-\Vert B^{-1}\Vert ^{-1},\Vert B^{-1}\Vert ^{-1})$ around 0. Then [6,  Thm. 4.7] with $\beta _T = 1/\left\Vert B^{-1}\right\Vert$ therein implies that $\mathrm{{i}}{\mathbb {R}}\subseteq \rho ({\mathcal {A}})$. $\square$

## Application to Damped Wave Equations in ${\mathbb {R}}^d$ with Unbounded Damping

In this section we use the results obtained in Sect. 3 to derive new spectral enclosures for linearly damped wave equations with non-negative possibly singular and/or unbounded damping *a* and potential *q*.

Our result covers a new class of unbounded dampings which are *p*-subordinate to $-\Delta +q$, a notion going back to [18,   §.7.1], [20,   §5.1], cf. [29,   Sect. 3].

### Theorem 7.1

Let ${\mathbf {t}}$ be a quadratic pencil of sesquilinear forms given by

$$\begin{aligned} {\mathbf {t}}(\uplambda ){:}{=}{\mathbf {t}}_0+2\uplambda {\mathbf {a}}+\uplambda ^2, \quad {\text {dom}}{\mathbf {t}}(\uplambda ){:}{=}{\text {dom}}{\mathbf {t}}_0, \quad \uplambda \in {\mathbb {C}}, \end{aligned}$$

where ${\mathbf {t}}_0$ and ${\mathbf {a}}$ are densely defined sesquilinear forms in ${\mathcal {H}}$ such that ${\mathbf {t}}_0$ is closed, ${\mathbf {t}}_0\ge \kappa _0\ge 0$, ${\mathbf {a}}\ge \alpha _0\ge 0$ and ${\text {dom}}{\mathbf {t}}_0\subseteq {\text {dom}}{\mathbf {a}}$. Suppose that there exist $\kappa \le \kappa _0$ and $p\in (0,1)$ such that ${\mathbf {a}}$ is *p*-form-subordinate with respect to ${\mathbf {t}}_0-\kappa \ge 0$, i.e. there is $C_{p}>0$ with

7.1 $$\begin{aligned} {\mathbf {a}}[f]\le C_{p}\big (({\mathbf {t}}_0-\kappa )[f]\big )^p \big (\left\Vert f\right\Vert ^2\big )^{1-p}, \quad f\in {\text {dom}}{\mathbf {t}}_0. \end{aligned}$$

**Fig. 2:**
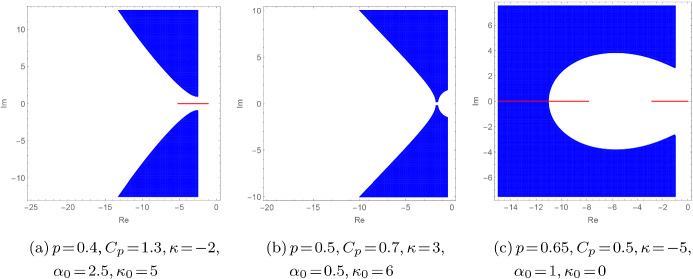
Enclosures for $\sigma (T) \!{\setminus }\! {\mathbb {R}}$ in Theorem 7.1 (i) (blue solid regions) and for $\sigma (T)\cap {\mathbb {R}}$ in Theorem 7.1 (ii)-(v) (red intervals in ${\mathbb {R}}$ in (a), (c), empty in (b)) (color figure online)

Then the family ${\mathbf {t}}$ is holomorphic of type (a). If $\,T$ denotes the associated holomorphic family of type (B), then

$$\begin{aligned} \sigma (T) \subseteq W_\Psi (T) \subseteq \big \{ z\!\in \!{\mathbb {C}}\!: {\text {Re}}z\le 0 \big \} \end{aligned}$$

and the following more precise spectral enclosures hold:

(i) The non-real spectrum of $\,T$ is contained in
  $$\begin{aligned} \sigma (T){\setminus } {\mathbb {R}}\subseteq W_\Psi (T) {\setminus } {\mathbb {R}}\subseteq \! \bigg \{&z\!\in \!{\mathbb {C}}\!: {\text {Re}}z\le -\alpha _0, \, |z| \ge \sqrt{\kappa _0} , \\&\left|{\text {Im}}z\right|\!\ge \! \sqrt{\max \!\Big \{0,C_{p}^{-\frac{1}{p}}\!\left|{\text {Re}}z\right|^\frac{1}{p}\!\!-\!\left|{\text {Re}}z\right|^2\!\!+\!\kappa \Big \}}\bigg \}; \end{aligned}$$
(ii) if $\,p\!<\!\frac{1}{2}$ or if $\,p\!=\!\frac{1}{2}$ and $C_{\frac{1}{2}}\! <\!1$ or if $p=\frac{1}{2}$ and $C_\frac{1}{2} = 1$ and $\kappa >0$, the real spectrum of $\,T$ satisfies either
  $$\begin{aligned} \sigma (T)\cap {\mathbb {R}}= \emptyset \quad \text{ or } \quad \sigma (T) \cap {\mathbb {R}}\subseteq [s^-,s^+ ], \end{aligned}$$
  if $\,p \!>\! \frac{1}{2}$ or if $\,p\!=\!\frac{1}{2}$ and $C_{\frac{1}{2}}\!>\!1$ or if $p=\frac{1}{2}$ and $C_\frac{1}{2} = 1$ and $\kappa \le 0$, the real spectrum of $\,T$ satisfies either
  $$\begin{aligned} \sigma (T)\cap {\mathbb {R}}\subseteq (-\infty , r^+] \cup [s^-\!, s^+] \ \ \text{ or } \ \ \sigma (T) \cap {\mathbb {R}}\subseteq (-\infty ,s^+], \end{aligned}$$
  where $\infty<r^+< s^- \!\le \! s^+ \!\le \! 0$ depend on *p*, $C_p$, $\kappa _0$ and $\kappa$;
(iii) if $\kappa =0$ and $p < \frac{1}{2}$, then
  $$\begin{aligned} \quad&\sigma (T)\cap {\mathbb {R}}= \emptyset \qquad \qquad \qquad \qquad \qquad \qquad \qquad \qquad \qquad \qquad \qquad \qquad \qquad \quad \,\, \text{ if } (C_p^2)^{\frac{1}{1-2p}} \!<\! \kappa _0, \\&\sigma (T)\cap {\mathbb {R}}\subseteq \!\!\Big [ \!-\!C_pt_0^p\!-\!\sqrt{C_{p}^pt_0^{2p}\!-\!t_0 }, -C_p\kappa _0^p \!+\!\sqrt{C_p^2\kappa _0^{2p}\!-\!\kappa _0}\Big ) \Big ]\; \text{ if } (C_p^2)^{\frac{1}{1-2p}} \!\ge \! \kappa _0, \end{aligned}$$
  where $$t_0:= \max \big \{ \big ( 4C_p^2p(1\!-\!p) \big )^{-\frac{1}{2p-1}}\!\!,\kappa _0 \big \}$$;
(iv) if $\kappa =0$ and $p= \frac{1}{2}$, then
  $$\begin{aligned}&\sigma (T)\cap {\mathbb {R}}= \emptyset \ \text{ if } C_{\frac{1}{2}}\!<\!1 \text{ and } \kappa _0 \!>\! 0, \\&\sigma (T) \cap {\mathbb {R}}\subseteq \{0\} \ \text{ if } C_\frac{1}{2} \!<\!1 \text{ and } \kappa _0 \!=\! 0, \\&\sigma (T)\cap {\mathbb {R}}\subseteq \!\Big (\!\!-\!\infty ,-\Big (C_\frac{1}{2} \!-\!\sqrt{C_\frac{1}{2}^2 \!-\! 1}\Big ) \kappa _0^{\frac{1}{2}} \Big ] \ \text{ if } C_{\frac{1}{2}} \!\ge \! 1; \end{aligned}$$
(v) if $\kappa =0$ and $p> \frac{1}{2}$, then
  $$\begin{aligned}&\sigma (T)\cap {\mathbb {R}}\subseteq \Big (\!\!-\!\infty , -C_pt_0^p+\sqrt{C_{p}^2t_0^{2p}\!-\!t_0 }\,\Big ]&\ \text{ if } \kappa _0 > 0, \\&\sigma (T)\cap {\mathbb {R}}\subseteq \Big (\!\!-\!\infty , -C_pt_0^p+\sqrt{C_{p}^2t_0^{2p}\!-\!t_0 }\,\Big ] \cup \{0\}&\ \text{ if } \kappa _0 = 0, \end{aligned}$$
  where $t_0:= \max \big \{ \big ( 4C_p^2p(1\!-\!p) \big )^{-\frac{1}{2p-1}}\!\!,\kappa _0 \big \}$. Figure 2 shows the different shapes of the enclosing regions in Theorem 7.1 depending on the parameters.

### Remark 7.2

If (7.1) holds with $p=0$, then ${\mathbf {a}}$ is bounded and $\left\Vert {\mathbf {a}}\right\Vert \le C_p= C_0$. In this case, the spectrum of *T* lies in a strip to the left of the imaginary axis; more precisely, the non-real spectrum of *T* satisfies

$$\begin{aligned} \sigma (T){\setminus }{\mathbb {R}}\subseteq \left\{ z \in {\mathbb {C}}:-C_0 \le {\text {Re}}z \le -\alpha _0, \, |z| \ge \sqrt{\kappa _0} \right\} , \end{aligned}$$

while the real spectrum satisfies

$$\begin{aligned} \sigma (T) \cap {\mathbb {R}}{\left\{ \begin{array}{ll} = \emptyset &{} \quad \mathrm{if } \,\, C_0^2 < \kappa _0, \\ \subseteq [-C_0 - \sqrt{C_0^2-\kappa _0}, -C_0 + \sqrt{C_0^2-\kappa _0}] &{} \quad \mathrm{if } \,\, C_0^2 \ge \kappa _0; \end{array}\right. } \end{aligned}$$

notice that the latter corresponds to Theorem 7.1 (iii) with $p=0$.

### Proof of Theorem 7.1

Clearly, ${\mathbf {t}}$ is holomorphic. For arbitrary $\varepsilon >0$, applying Young’s inequality to (7.1), we obtain

$$\begin{aligned} \begin{aligned} {\mathbf {a}}[f]&\le \left( \frac{\varepsilon }{p}\right) ^p \!\!\! \big (({\mathbf {t}}_0-\kappa )[f]\big )^p \left( \frac{p}{\varepsilon }\right) ^p C_{p}\big (\left\Vert f\right\Vert ^2\big )^{1-p}\\&\le \varepsilon \big (({\mathbf {t}}_0-\kappa )[f]\big )+ (1\!-\!p)\left( \frac{p}{\varepsilon }\right) ^\frac{p}{1-p} C_p^\frac{1}{1-p} \left\Vert f\right\Vert ^2 \end{aligned} \end{aligned}$$

for all $f\in {\text {dom}}{\mathbf {t}}_0$, i.e. ${\mathbf {a}}$ is ${\mathbf {t}}_0$-bounded with relative bound 0. Hence, for each $\uplambda \in {\mathbb {C}}$, the form ${\mathbf {t}}(\uplambda )$ is densely defined, sectorial and closed, see e.g. [17,   Thm. VI.1.33]. This shows that ${\mathbf {t}}$ is a holomorphic family of type (a). Since all enclosing sets in Theorem 7.1 are closed and

$$\begin{aligned} \sigma (T) \subseteq W_{\Psi } (T)=W_{\Psi } ({\mathbf {t}}) = \overline{W({\mathbf {t}})} \end{aligned}$$

by Theorem 3.3 with $k=2$ and $\mu \in {\mathbb {C}}$ arbitrary, it suffices to show that $W({\mathbf {t}}){\setminus } {\mathbb {R}}$ and $W({\mathbf {t}})\cap {\mathbb {R}}$ satisfy the claimed enclosures.

Let $\uplambda _0\in W({\mathbf {t}})$, i.e. there exists $f\in {\text {dom}}{\mathbf {t}}_0$, $\left\Vert f\right\Vert =1$, with ${\mathbf {t}}(\uplambda _0)[f]=0$. Taking real and imaginary part in this equation, we conclude that

7.2 $$\begin{aligned} {\mathbf {t}}_0[f]+2{\text {Re}}\uplambda _0\,{\mathbf {a}}[f]+({\text {Re}}\uplambda _0)^2-({\text {Im}}\uplambda _0)^2&=0, \end{aligned}$$

7.3 $$\begin{aligned} 2{\text {Im}}\uplambda _0\,{\mathbf {a}}[f]+2{\text {Re}}\uplambda _0{\text {Im}}\uplambda _0&=0. \end{aligned}$$

First assume that ${\uplambda _0 \!\in }W({\mathbf {t}}){\setminus }{\mathbb {R}}$. Dividing (7.3) by $2{\text {Im}}\uplambda _0$ ($\ne 0$) and inserting this into (7.2), we find

$$\begin{aligned}&{\text {Re}}\uplambda _0=-{\mathbf {a}}[f]\le -\alpha _0\le 0, \nonumber \\&\left|\uplambda _0\right|^2=({\text {Im}}\uplambda _0)^2+({\text {Re}}\uplambda _0)^2={\mathbf {t}}_0[f] \ge \kappa _0. \end{aligned}$$

Using these relations and assumption (7.1), we can further estimate

$$\begin{aligned} ({\text {Im}}\uplambda _0)^2 ={\mathbf {t}}_0[f] - |{\text {Re}}\uplambda _0|^2 \ge \max \{ 0, {C_p^{-\frac{1}{p}} } \left|{\text {Re}}\uplambda _0\right|^\frac{1}{p} - |{\text {Re}}\uplambda _0|^2 +\kappa \}, \end{aligned}$$

and hence $\uplambda _0 \!\in W({\mathbf {t}})\!{\setminus }\!{\mathbb {R}}$ satisfies all three claimed inequalities in (i).

Now assume that $\uplambda _0 \!\in \!W({\mathbf {t}})\cap {\mathbb {R}}$. Then ${\mathbf {a}}[f]^2\!-\!{\mathbf {t}}_0[f]\!\ge \!0$ and thus, in particular, ${\mathbf {a}}[f] \!\ge \! \max \{\alpha _0,\sqrt{\kappa _0}\}$. Moreover, since ${\text {Im}}\uplambda _0\!=\!0$, equality (7.3) trivially holds and (7.2) implies

7.4 $$\begin{aligned} \uplambda _0 = -{\mathbf {a}}[f]\pm \sqrt{{\mathbf {a}}[f]^2-{\mathbf {t}}_0[f]}\le 0 \end{aligned}$$

because ${\mathbf {t}}_0 \!\ge \! 0$. This, together with ${\mathbf {a}}\!\ge \! \alpha _0$ and assumption (7.1), yields that

7.5 $$\begin{aligned} \max \big \{\alpha _0^2,\kappa _0\big \} \le \max \{ \alpha _0^2, {\mathbf {t}}_0[f]\} \le {\mathbf {a}}[f]^2 \le C_p^2 \big (({\mathbf {t}}_0-\kappa )[f]\big )^{2p}. \end{aligned}$$

If we define

$$\begin{aligned}&d(x):= C_p^{-\frac{1}{p}} x^{\frac{1}{2p}} \!-\!x \!+\! \kappa , \quad x\!\in \! [0,\infty ),\quad D_{\le 0} := \big \{ x \!\in \! [\kappa _0,\infty ): d(x) \le 0\big \}, \end{aligned}$$

then it is easy to see that ${\mathbf {t}}_0[f] \!\in \! D_{\le 0}$;

in particular, $D_{\le 0} = \emptyset$ implies $W({\mathbf {t}}) \cap {\mathbb {R}}=\emptyset$. An elementary analysis shows that *d* is either identically zero, has no zero, one simple zero or two (possibly coinciding) zeros on $[0,\infty )$, which we denote by $x_+$ and $x_-\le x_+$, respectively, if they exist. Then

7.6 $$\begin{aligned} p<&\frac{1}{2} \text{ or } p = \frac{1}{2}, C_\frac{1}{2} < 1 \text{ or } p = \frac{1}{2}, C_\frac{1}{2} = 1, \kappa >0 \nonumber \\&\implies D_{\le 0} \!=\! \emptyset \text{ or } D_{\le 0} \text{ is } \text{ bounded }, \ D_{\le 0} \!=\! [ \kappa _0,x_+] \text{ or } D_{\le 0}\!=\![x_-,x_+], \end{aligned}$$

7.7 $$\begin{aligned} p>&\frac{1}{2} \text{ or } p = \frac{1}{2}, C_\frac{1}{2} > 1 \text{ or } p = \frac{1}{2}, C_\frac{1}{2} = 1, \kappa \le 0 \nonumber \\&\implies \!\! D_{\le 0} \!\ne \! \emptyset \text{ is } \text{ unbounded },\!\!\ D_{\le 0}\!=\![\kappa _0,\infty ) \text{ or } D_{\le 0} \!=\! [x_+, \infty )\nonumber \\&\text{ or } D_{\le 0}\!=\![\kappa _0,x_-]\!\cup \![x_+,\infty ). \end{aligned}$$

Which case prevails for fixed $p \!\in \! [0,1)$ can be characterised by means of inequalities involving the constants $\kappa _0$, $\kappa$ and $C_p$. For estimating $\uplambda _0$ in (7.4) while respecting the restrictions in (7.5), we consider the functions

$$\begin{aligned} f_\pm (s,t):= -s \pm \sqrt{s^2 \!-\! t}, \quad s\!\in \![\alpha _0,\infty ), \ t\!\in \! [\kappa _0,\infty ), \ t \!\le \! s^2 \!\le \! C_p^2(t-\kappa )^{2p}. \end{aligned}$$

It is easy to check that $f_+$ is monotonically increasing in *s* and monotonically decreasing in *t*, while $f_-$ is monotonically decreasing in *s* and monotonically increasing in *t* and hence, since $s \le C_p(t-\kappa )^{p}$,

7.8 $$\begin{aligned} \begin{aligned} f_+(s,t)&\le f_+(C_p(t-\kappa )^p,t) {=}{:}g_+(t), \\ f_-(s,t)&\ge f_-(C_p(t-\kappa )^p,t) {=}{:}g_-(t). \end{aligned} \end{aligned}$$

Now we distinguish the two qualitatively different cases (7.6) and (7.7). To obtain the claimed enclosures for $W({\mathbf {t}})\cap {\mathbb {R}}$, we use (7.5), (7.4) and (7.8) to conclude that $g_-(t) \le \uplambda _0 \le g_+(t)$ for some $t \in D_{\le 0}$.

If (7.6) holds, there are the following two possibilities:

(1) If *d* has no zeros on $[0,\infty )$ or if *d* has at least one zero and $x_+ \!<\!\kappa _0$, then $D_{\le 0} =\emptyset$ and thus

$$\begin{aligned} W({\mathbf {t}})\cap {\mathbb {R}}= \emptyset . \end{aligned}$$

(2) If *d* has at least one zero $x_+$ and $x_+ \ge \kappa _0$, then $D_{\le 0}$ is one bounded interval and

$$\begin{aligned} W({\mathbf {t}})\!\cap {\mathbb {R}}\subseteq \!\big [s^-\!, s^+\big ], \quad&s^- \!{:}{=}\!\! \min _{t\in D_{\le 0} }g_-(t), \quad s^+ \!{:}{=}\!\! \max _{t\in D_{\le 0} }g_+(t); \end{aligned}$$

here if *d* has only one zero $x_+$ or if *d* has two zeros $x_\pm$ and $x_-<\kappa _0$, then $D_{\le 0} = [\kappa _0,x_+]$ and if *d* has two zeros and $x_-\ge \kappa _0$, then $D_{\le 0} =[x_-,x_+]$.

If (7.7) holds, there are the following two possibilities:

(3) If *d* has two zeros $x_\pm$ on $[0,\infty )$ and $x_- \!\ge \! \kappa _0$, then $D_{\le 0}\!=\! [\kappa _0,x_-] \cup [x_+,\infty )$ and we obtain

$$\begin{aligned} W({\mathbf {t}})\cap {\mathbb {R}}\!\subseteq \! \big (\!-\!\infty , r^+ \big ] \! \cup \! \big [ s^-\!,s^+ \big ], \ \&r^+ \!{:}{=}\!\! \max _{t \in [x_+,\infty )} g_+(t), \ s^+ \!{:}{=}\!\!\! \max _{t\in [\kappa _0,x_-]}g_+(t), \\[-1mm]&s^- \!{:}{=}\!\!\! \min _{t\in [\kappa _0,x_-]}g_-(t) ; \end{aligned}$$

here $g_+$ attains a maximum on $[x_+,\infty )$ since $g_+(t)$ tends to $-\infty$ as $t\rightarrow \infty$, and analogously in the next case.

(4) If *d* has either at most one zero $x_+$ or two zeros $x_\pm$ on $[0,\infty )$ and $x_- < \kappa _0$, then $D_{\le 0} = [\max \{\kappa _0, x_+\},\infty )$ and we conclude that

$$\begin{aligned} W({\mathbf {t}})\cap {\mathbb {R}}\subseteq \big (-\infty , s^+\big ], \quad s^+ \!{:}{=}\! \max _{t \in [\max \{\kappa _0, x_+\},\infty )} g_+(t). \end{aligned}$$

This proves claim (ii).

Claim (iv) for $\kappa \!=\!0$ and $p\!=\! \frac{1}{2}$ follows from cases (1), (2) and (4) above if we note that then $d(x)=(C_{\frac{1}{2}}^{-2}\!-\!1)x$, $x\!\in \![0,\infty )$, is either identically zero or has the only zero $x_+\!=\!0$ and, for case (4), $g_+(t)\!=\!-t^{\frac{1}{2}} \big (C_{\frac{1}{2}}\!+\! \sqrt{C_{\frac{1}{2}}^2\!-\!1}\big )$ is montonically decreasing so that $s^+=g_+(\kappa _0)$.

Finally, if $\kappa \!=\!0$ and $p\!\ne \! \frac{1}{2}$, the function *d* has the two zeros $x_-\!=\!0$ and $x_+ \!=\! (C_p^2)^{\frac{1}{1-2p}}$ on $[0,\infty )$, and the respective bounds $r^+$, $s^\pm$ above can be determined explicitly to deduce claims (iii) and (v). More precisely, claim (iii) follows from cases (1) and (2) if we note that, in (2), $D_{\le 0} = [\kappa _0, x_+]$, $g_+$ is monotonically decreasing on $[0, x_+]$ and $g_-$ attains its minimum on $[0,x_+]$ at $t=\big ( 4C_p^2p(1\!-\!p) \big )^{-\frac{1}{2p-1}}$. Claim (v) follows from cases (4) if $\kappa _0>0$ and (3) if $\kappa _0=0$; note that, for $\kappa \!=\!0$, case (3) where $p\!>\!1/2$ can only occur if $\kappa _0\!=\!0$. In both cases, we use that $g_+$ attains its maximum on $[x_+ ,\infty )$ at $t=\big ( 4C_p^2p(1\!-\!p) \big )^{-\frac{1}{2p-1}}$. $\square$

### Remark 7.3

If (7.1) holds with $\kappa \le \kappa _0$ and $p\in [0,1)$, then it holds for every $q\in (p,1)$ with $\kappa _1 \le \kappa$ such that $\kappa _1 < \kappa _0$.

Indeed, then ${\mathbf {t}}_0\!-\!\kappa \! \le \! {\mathbf {t}}_0\!-\!\kappa _1$ and ${\mathbf {t}}_0\! -\!\kappa _1 \!\ge \! \kappa _0\!-\!\kappa _1 \!>\!0$ which implies that $(\Vert f\Vert ^2)^{q-p} \!\le \! (\kappa _0\!-\!\kappa _1)^{p-q} \big (({\mathbf {t}}_0\!-\!\kappa _1)[f] \big )^{q-p}$, $f\!\in \! {\text {dom}}{\mathbf {t}}_0$. Hence (7.1) holds with *q*, $\kappa _1$ and $C_q=C_p (\kappa _0\!-\!\kappa _1)^{p-q}$.

### Remark 7.4

As a special case of Theorem 7.1 we obtain the enclosure for the non-real spectrum proved in [15,  Thm. .2, Part 5] (where the damping was only assumed to be accretive) and we considerably improve the enclosure for the real spectrum therein since we obtain that the latter is, in fact, empty. The assumption in [15,  Thm. 3.2, Part 5] is that

7.9 $$\begin{aligned} \nu := \sup _{f\in {\text {dom}}{\mathbf {t}}_0{\setminus }\{0\}} \frac{2{\mathbf {a}}[f]}{{\mathbf {t}}_0[f]^{1/2}\Vert f\Vert } \in (0,2). \end{aligned}$$

The parameters $a_0$, $\beta$ and $\nu$ in [15,  (5) and p. 3] correspond to the following special choices in Theorem 7.1 and assumption (7.1):

$$\begin{aligned} p=\frac{1}{2}, \quad C_{\frac{1}{2}} = \frac{\nu }{2}, \quad \kappa =0, \quad \kappa _0 = a_0^2 >0, \quad \alpha _0 = \frac{\beta }{2}. \end{aligned}$$

Under the assumption (7.9) made in [15,  Thm. 3.2, Part ], Theorem 7.1 (i) yields the spectral enclosure

$$\begin{aligned} \sigma (T) {\setminus } {\mathbb {R}}\subseteq \! \bigg \{ z\!\in {\mathbb {C}}: {\text {Re}}z\!\le \!-\frac{\beta }{2}, \, \left|z\right| \!\ge \! a_0, \, \, \left|{\text {Im}}z\right|\!\ge \! \sqrt{\frac{4}{\nu ^2}\!-\!1\,}\left|{\text {Re}}z\right| \bigg \}. \end{aligned}$$

This enclosure is the same as in [15,  Thm. 3.2, Part ]. However, since $\nu \!<\!2$ is equivalent to $C_{\frac{1}{2}} \!<\!1$, the enclosure $\sigma (T)\cap {\mathbb {R}}\subseteq (-\infty ,-\frac{a_0}{\nu }-\frac{4a_0}{\nu ^3}]$ in [15,  Thm. 3.2, Part 5] is considerably improved by Theorem 7.1 (iv) to

$$\begin{aligned} \sigma (T)\cap {\mathbb {R}}= \emptyset . \end{aligned}$$

### Remark 7.5

In the second case in Theorem 7.1 (ii), i.e. if $p \!>\! \frac{1}{2}$ or $p \!=\! \frac{1}{2}$, $C_\frac{1}{2} \!>\! 1$ or $p \!=\! \frac{1}{2}$, $C_\frac{1}{2} \!=\! 1$, $\kappa \!\le \! 0$, the set $W({\mathbf {t}})\cap (-\infty ,0]$ used to enclose the spectrum can, indeed, be unbounded if so is ${\mathbf {t}}_0$.

In fact, if $W({\mathbf {t}}_0) \!=\! [\kappa _0,\infty )$, we can choose ${\mathbf {a}}\!=\!C_p({\mathbf {t}}_0-\kappa )^p$. Then there exist $f_n\!\in \!{\text {dom}}{\mathbf {t}}_0$, $\left\Vert f_n\right\Vert \!=\!1$, with ${\mathbf {t}}_0[f_{n}] \!\ge \! n$ for $n\!\in \!{\mathbb {N}}$. The conditions on *p*, $C_p$ and $\kappa$ ensure, comp. (7.7), that $C_p^2({\mathbf {t}}_0[f_{n}]-\kappa )^{2p}-{\mathbf {t}}_0[f_{n}]\ge 0$ for sufficiently large $n\in \!{\mathbb {N}}$ and thus

$$\begin{aligned} W({\mathbf {t}})\cap (-\infty ,0]\ni \uplambda _0\!=\!-{\mathbf {t}}_0[f_n]^p\!-\!\sqrt{{\mathbf {t}}_0[f_n]^{2p}\!-\!{\mathbf {t}}_0[f_n]}\le -{\mathbf {t}}_0[f_n]^p \le -n^p \rightarrow -\infty , \end{aligned}$$

and hence $$\inf \left( W({\mathbf {t}})\cap (-\infty ,0]\right) =-\infty $$.

In the next example we apply Theorem 7.1 to linearly damped wave equations with possibly unbounded and/or singular damping.

### Example 7.6

Let ${\mathcal {H}}=L^2({\mathbb {R}}^d)$ with $d\ge 3$ and *a*, $q\in L^1_{{\text {loc}}}({\mathbb {R}}^d)$, $a \ne 0$ and $a,q\ge 0$ almost everywhere. If ${\text {dom}}a^\frac{1}{2}$ and ${\text {dom}}q^\frac{1}{2}$ denote the maximal domains of the multiplication operators $a^\frac{1}{2}$ and $q^\frac{1}{2}$ in $L^2({\mathbb {R}}^d)$, respectively, we define the quadratic forms ${\mathbf {a}}$ and ${\mathbf {t}}_0$ in $L^2({\mathbb {R}}^d)$ by

$$\begin{aligned} \begin{aligned} {\mathbf {a}}[f]&{:}{=}\int _{{\mathbb {R}}^d}a\left|f\right|^2\mathrm{d}x,&\qquad {\text {dom}}{\mathbf {a}}&{:}{=}{\text {dom}}a^\frac{1}{2}, \\ {\mathbf {t}}_0[f]&{:}{=}\int _{{\mathbb {R}}^d}\left|\nabla f\right|^2\mathrm{d}x+\int _{{\mathbb {R}}^d}q\left|f\right|^2\mathrm{d}x,&\qquad \qquad \, {\text {dom}}{\mathbf {t}}_0&{:}{=}H^1({\mathbb {R}}^d)\cap {\text {dom}}q^\frac{1}{2}. \end{aligned} \end{aligned}$$

Suppose that, for almost all $x\in {\mathbb {R}}^d$,

7.10 $$\begin{aligned} a(x)\le \sum _{j=1}^n\left|x-x_j\right|^{-t}+ u(x) + v(x), \qquad v(x) \le c_1 q(x)^r+c_2, \end{aligned}$$

where $u \in L^s ({\mathbb {R}}^d)$ with $s>d/2$, $v\!\in \!L^1_{{\text {loc}}}({\mathbb {R}}^d)$, $t\!\in \![0,2)$, $n\!\in \!{\mathbb {N}}_{0}$, $x_j\!\in \!{\mathbb {R}}^d$ for $j\!=\!1,\dotsc ,n$, $c_1$, $c_2\!\ge \!0$ and $r\!\in \![0,1)$. Then ${\mathbf {a}}$, ${\mathbf {t}}_0$ are closed, ${\mathbf {a}}$, ${\mathbf {t}}_0\ge 0$ and, without further assumptions, we only know that $\alpha _0 \ge 0$, $\kappa _0 \ge 0$ in Theorem 7.1. In order to verify (7.1), let $f\in {\text {dom}}{\mathbf {t}}_0$ with $\left\Vert f\right\Vert =1$. By Hölder’s and Hardy’s inequality, for $1\le j\le n$,

7.11 $$\begin{aligned} \begin{aligned} \int _{{\mathbb {R}}^d}\!\!\left|x\!-\!x_j\right|^{-t}\left|f\right|^2 \mathrm{d}x&\le \left( \int _{{\mathbb {R}}^d}\!\!\left|x\!-\!x_j\right|^{-2}\left|f\right|^2\mathrm{d}x\right) ^\frac{t}{2} \!\le \! \frac{2^t}{(d-2)^t}\left\Vert \nabla f\right\Vert ^t. \end{aligned} \end{aligned}$$

Further, by Gagliardo-Nirenberg-Sobolev’s inequality, there exists a constant $G_d{>}0$ depending only on the dimension *d* such that

$$\begin{aligned} \left\Vert f\right\Vert _{L^{2^*}({\mathbb {R}}^d)} \le G_d \left\Vert \nabla f\right\Vert , \quad f\in H^1 ({\mathbb {R}}^d), \quad 2^*{:}{=}\frac{2d}{d-2}, \end{aligned}$$

where $2^*\!>\!2$ is the critical Sobolev exponent for the embedding $H^1 ({\mathbb {R}}^d) \!\hookrightarrow \! L^{2^*}({\mathbb {R}}^d)$. Since $d/s \!\in \! (0,2)$, we can use Hölder’s inequality with three terms to estimate

$$\begin{aligned} \int _{{\mathbb {R}}^d} u |f|^2 \mathrm{d}x \le \left\Vert u\right\Vert _{L^s({\mathbb {R}}^d)} \left( \int _{{\mathbb {R}}^d} |f|^{\frac{d}{s} \frac{2s}{d-2}} \mathrm{d}x\right) ^\frac{d-2}{2s} \left( \int _{{\mathbb {R}}^d} |f|^{\left( 2-\frac{d}{s}\right) \frac{2s}{2s-d}} \mathrm{d}x\right) ^\frac{2s-d}{2s}. \end{aligned}$$

This inequality, together with the relations

$$\begin{aligned} \frac{d}{s} \frac{2s}{d-2} = 2^*, \quad \frac{d-2}{2s} = \frac{d}{2^*s}, \quad \left( 2-\frac{d}{s}\right) \frac{2s}{2s-d} = 2, \end{aligned}$$

and $\left\Vert f\right\Vert =1$, yields that

7.12 $$\begin{aligned} \int _{{\mathbb {R}}^d} u |f|^2 \mathrm{d}x \le \left\Vert u\right\Vert _{L^s({\mathbb {R}}^d)} \left\Vert f\right\Vert _{L^{2^*} ({\mathbb {R}}^d)}^\frac{d}{s} \le \left\Vert u\right\Vert _{L^s({\mathbb {R}}^d)} G_d^\frac{d}{s} \left\Vert \nabla f\right\Vert ^\frac{d}{s}. \end{aligned}$$

Next the bound on *v* in (7.10) with $r\in [ 0,1)$, Hölder’s inequality with $1/r\in (1,\infty ]$, $1/(1-r)\in [1,\infty )$ and $\left\Vert f\right\Vert =1$ give

7.13 $$\begin{aligned} \int _{{\mathbb {R}}^d} v |f|^2 \mathrm{d}x \le c_1\int _{{\mathbb {R}}^d}q^r\left|f\right|^2\mathrm{d}x + c_2 \le c_1\left( \int _{{\mathbb {R}}^d}q\left|f\right|^2\mathrm{d}x\right) ^r + c_2. \end{aligned}$$

Combining the inequalities (7.11), (7.12) and (7.13), we arrive at

7.14 $$\begin{aligned} \begin{aligned} {\mathbf {a}}[f] \!&\le \! \frac{n2^t}{(d\!-\!2)^t}\left\Vert \nabla f\right\Vert ^t \!\!+\! \left\Vert u\right\Vert _{L^s({\mathbb {R}}^d)} G_d^\frac{d}{s} \left\Vert \nabla f\right\Vert ^\frac{d}{s} \!+\! c_1\left( \int _{{\mathbb {R}}^d} \!q\left|f\right|^2\mathrm{d}x\right) ^{\!r}\!\!\!+\!c_2 \\&=: \alpha _1 (\left\Vert \nabla f\right\Vert ^2)^{\frac{t}{2}} \!\!+\! \alpha _2 (\left\Vert \nabla f\right\Vert ^2)^{\frac{d}{2s}} \!+\! \alpha _3 \left( \int _{{\mathbb {R}}^d} \!q\left|f\right|^2\mathrm{d}x\right) ^{r} \!+\! \alpha _4. \end{aligned} \end{aligned}$$

In order to further bound (7.14), we estimate $\alpha _1 x_1^{p_1} \!\!+\! \alpha _2 x_2^{p_2} \!+\! \alpha _3 x_3^{p_3} \!+\! \alpha _4$ with $x_i \!\ge \! 0$, $p_i \!\in \! [0,1)$, $i\!=\!1,2,3$, and $\alpha _i \!\ge \! 0$, $i\!=\!1,2,3,4$; note that $x_1\!=\!x_2=\Vert \nabla f\Vert ^2$ in (7.14). If we set $p \!{:}{=}\! \max \{p_1,p_2,p_3\}$ and maximise $\delta (x) \!{:}{=}\! x^{p_i} \!-\! x^p\!$, $x\in [0,1]$, $i\!=\!1,2,3$, we find that

7.15 $$\begin{aligned} x_i^{p_i} \!\le \! x_i^{p} \!+\!\delta _i, \quad \delta _i {:}{=}{\left\{ \begin{array}{ll} 0 &{} \text{ if } p_i = p, \\ \frac{p-p_i}{p} \left( \frac{p_i}{p}\right) ^\frac{p_i}{p-p_i} &{} \text{ if } p_i < p, \end{array}\right. } \quad i=1,2,3. \end{aligned}$$

If $$\max \{\alpha _1, \alpha _2, \alpha _3\} \ne 0$$, then

7.16 $$\begin{aligned} \gamma _p {:}{=}\alpha _1 (1 \!+\! \delta _1) \!+\! \alpha _2 (1 \!+\! \delta _2) \!+\! \alpha _3 (1 \!+\! \delta _3) \!+\! \alpha _4 \ne 0. \end{aligned}$$

If we use (7.15), the concavity of $x\!\mapsto \! x^{p}$ on $[0,\infty )$ and $x_1\!=\!x_2$, we obtain

$$\begin{aligned}&\alpha _1 x_1^{p_1} \!\!+\! \alpha _2 x_2^{p_2} \!+\! \alpha _3 x_3^{p_3} \!+\! \alpha _4 \, \le \,\alpha _1 (x_1^p \!+\! \delta _1) \!\!+\! \alpha _2 (x_2^{p} \!+\!\delta _2 ) \!+\! \alpha _3 (x_3^p \!+\! \delta _3) \!+\! \alpha _4 \\&\quad = \gamma _p \Big ( \frac{\alpha _1}{\gamma _p } x_1^{p} \!\!+\! \frac{\alpha _2}{\gamma _p } x_2^p + \frac{\alpha _3}{\gamma _p} x_3^p\!\!+\! \frac{\alpha _1\delta _1 + \alpha _2 \delta _2\!+\!\alpha _3\delta _3 + \alpha _4}{\gamma _p } \Big ) \\&\quad \le \gamma _p \Big ( \frac{\alpha _1}{\gamma _p } x_1 \!+\! \frac{\alpha _2}{\gamma _p } x_2 + \frac{\alpha _3}{\gamma _p} x_3 \!+\! \frac{\alpha _1\delta _1 + \alpha _2 \delta _2 \!+\!\alpha _3 \delta _3 + \alpha _4}{\gamma _p } \Big )^{p} \\&\quad = \gamma _p ^{1-p} \big ( (\alpha _1 + \alpha _2) x_1 \!+\! \alpha _3 x_3 + \alpha _1\delta _1 + \alpha _2 \delta _2 \!+\!\alpha _3 \delta _3 + \alpha _4 \big )^{p} \\&\quad \le \gamma _p ^{1-p} \max \{ \alpha _1 +\alpha _2, \alpha _3\}^{p} \Big ( x_1 \!+\! x_3 \!+\! \frac{\alpha _1\delta _1 + \alpha _2 \delta _2 \!+\!\alpha _3 \delta _3+\alpha _4}{\max \{ \alpha _1+\alpha _2, \alpha _3\}} \Big )^{p}. \end{aligned}$$

If $\max \{n,\left\Vert u\right\Vert _{L^s({\mathbb {R}}^d)},c_1\} \!\ne \! 0$, we can apply this estimate to (7.14) with $p_1 \!=\! t/2$, $p_2 \!=\! d/(2s)$, $p_3 \!=\! r$, $\delta _i$, $i\!=\!1,2,3$, as in (7.15) to obtain that ${\text {dom}}{\mathbf {t}}_0\subseteq {\text {dom}}{\mathbf {a}}$ and assumption (7.1) holds with the parameters

7.17 $$\begin{aligned} \begin{aligned}&p\!=\!\max \Big \{\frac{t}{2},\frac{d}{2s}, r\!\Big \}, \quad C_p \!=\! \gamma _p^{1-p} \max \Big \{ \frac{n2^t}{(d\!-\!2)^t} + \left\Vert u\right\Vert _{L^s({\mathbb {R}}^d)} G_d^{\frac{d}{s}},c_1 \Big \}^{\!p}\!\!, \\&\kappa \!=\!- \frac{ n2^t \delta _1 + (d-2)^t (\left\Vert u\right\Vert _{L^s({\mathbb {R}}^d)} G_{d}^{\frac{d}{s}} \delta _2 + c_1 \delta _3 + c_2)}{\max \{ n2^t \!+\! (d\!-\!2)^t \left\Vert u\right\Vert _{L^s({\mathbb {R}}^d)} G_d^{\frac{d}{s}},\,(d-2)^t c_1\}}, \end{aligned} \end{aligned}$$

where, according to (7.16),

$$\begin{aligned} \gamma _p = \frac{n2^t}{(d-2)^t} (1 \!+\! \delta _1) \!+\! \left\Vert u\right\Vert _{L^s({\mathbb {R}}^d)} G_d^\frac{d}{s} (1 \!+\! \delta _2) \!+\! c_1 (1 \!+\! \delta _3) \!+\! c_2. \end{aligned}$$

If $\max \{n,\left\Vert u\right\Vert _{L^s({\mathbb {R}}^d)},c_1\} \!=\! 0$, i.e. $n=0$, $u\equiv 0$ and $c_1=0$, then the damping $a=v$ is bounded, our assumption $a\ne 0$ implies $c_2>0$ and (7.1) trivially holds with $p=0$, $C_0=c_2 =\Vert a\Vert _\infty$ and $\kappa \le d=\kappa _0$ arbitrary.

The constants in (7.17) in the general case $\max \{n,\left\Vert u\right\Vert _{L^s({\mathbb {R}}^d)},c_1\} \!\ne \! 0$ simplify substantially if either $n\!=\!0$, $u\!\equiv \! 0$ or $v \!\equiv \! 0$. If e.g. two of *n*, *u* or *v* vanish, the constants *p*, $C_p$ and $\kappa$, which may be read off from (7.11), (7.12) or (7.13), are also obtained as special cases of (7.17). For instance,

$$\begin{aligned}&p=\frac{t}{2}, \quad C_{\frac{t}{2}} = \frac{n2^t}{(d-2)^t}, \quad \kappa = 0&\quad \text{ if }\, n\ne 0, u\equiv 0 \text{ and } v\equiv 0,\\&p=\frac{d}{2s}, \ C_\frac{d}{2s} = \left\Vert u\right\Vert _{L^s({\mathbb {R}}^d)} G_d^\frac{d}{s}, \ \kappa = 0&\quad \text{ if }\, n= 0, u\not \equiv 0 \text{ and } v\equiv 0,\\&p=r, \quad C_r = (c_1\!+\!c_2)^{1-r} c_1^r, \quad \kappa = -\frac{c_2}{c_1}&\quad \qquad \text{ if }\, n= 0, u\equiv 0 \text{ and } v\not \equiv 0, c_1\!>\!0; \end{aligned}$$

in (7.17) these are the 3 cases $\delta _1 = 0$ with $c_1=c_2=r=0$ and *s* sufficiently large such that $d/(2s)<r$, $\delta _2 = 0$ with $t=c_1=c_2=r=0$, and $\delta _3=0$ with $t=0$ and *s* sufficiently large, respectively. The cases where only one of *n*, *u* or *v* vanishes are similar and are left to the reader.

As a special case, we consider

$$\begin{aligned} a(x)=\left|x\right|^{k} \ \text{ with } k \in [0,2), \quad q(x)=\left|x\right|^2, \quad x\in {\mathbb {R}}^d. \end{aligned}$$

Here $\alpha _0\!=\!0$ and we can choose $\kappa _0 >0$ as the ground energy of the harmonic oscillator, cf. [25,  Sect. III.12], i.e.

$$\begin{aligned} \kappa _{0}=\inf _{f\in {\text {dom}}{\mathbf {t}}_0}\frac{{\mathbf {t}}_0[f]}{\left\Vert f\right\Vert ^2}=\frac{{\mathbf {t}}_0[f_0]}{\left\Vert f_0\right\Vert ^2}=d, \end{aligned}$$

where $f_0(x)=\exp (-\left|x\right|^2\!/2)$, $x\in {\mathbb {R}}^d$, is the (non-normalised) ground state of the harmonic oscillator. Moreover, in this special case *a* satisfies (7.10) with

$$\begin{aligned} n\!=\!0, \quad t\!=\!0, \quad u \equiv 0, \quad v \equiv a, \quad r = \frac{k}{2}, \quad c_1 = 1, \quad c_2=0, \end{aligned}$$

and by what was shown above, condition (7.1) holds with

$$\begin{aligned} p=\frac{k}{2}, \quad C_p=1, \quad \kappa =0. \end{aligned}$$

Hence the results in Theorem 7.1 (iii), (iv) and (v) yield that

$$\begin{aligned} \sigma (T) {\setminus }{\mathbb {R}}\subseteq \Big \{z\!\in \!{\mathbb {C}}:{\text {Re}}z\!\le \!0, \, \left|z\right|\!\ge \! \sqrt{d}, \, |{\text {Im}}z| \!\ge \! \sqrt{\max \{0,\left|{\text {Re}}z\right|^{\!\frac{2}{k} }\!\!-\!\left|{\text {Re}}z\right|^2\}}\Big \} \end{aligned}$$

and

$$\begin{aligned} \sigma (T) \cap {\mathbb {R}}{\left\{ \begin{array}{ll} = \emptyset &{} \text{ if } k\in [0,1), \\ \subseteq (-\infty ,-\sqrt{d}] &{} \text{ if } k=1, \\ \subseteq \!\Big (\!\!-\!\infty ,-\sqrt{t_0}^{k}\!+\!\sqrt{t_0^{k}\!-\!t_0 } \,\Big ] &{} \text{ if } k\in (1,2), \end{array}\right. } \end{aligned}$$

where in the latter case $t_0=\max \big \{ \big ( k(2-k) \big )^{-\frac{1}{k-1}},d\big \}$.

## Data Availability

Data sharing not applicable to this article as no datasets were generated or analysed during the current study.
